# A Numb–Mdm2 fuzzy complex reveals an isoform-specific involvement of Numb in breast cancer

**DOI:** 10.1083/jcb.201709092

**Published:** 2018-02-05

**Authors:** Ivan Nicola Colaluca, Andrea Basile, Lee Freiburger, Veronica D'Uva, Davide Disalvatore, Manuela Vecchi, Stefano Confalonieri, Daniela Tosoni, Valentina Cecatiello, Maria Grazia Malabarba, Chun-Jiun Yang, Masatsune Kainosho, Michael Sattler, Marina Mapelli, Salvatore Pece, Pier Paolo Di Fiore

**Affiliations:** 1 The FIRC Institute for Molecular Oncology Foundation, Milan, Italy; 2 Department of Health Sciences, Università degli Studi di Milano, Milan, Italy; 3 Department of Oncology and Hemato-Oncology, Università degli Studi di Milano, Milan, Italy; 4 Center for Integrated Protein Science Munich, Department of Chemistry, Technical University of Munich, Garching, Germany; 5 Institute of Structural Biology, Helmholtz Zentrum München, Neuherberg, Germany; 6 Program of Molecular Medicine, European Institute of Oncology, Milan, Italy; 7 Department of Experimental Oncology, European Institute of Oncology, Milan, Italy; 8 Structural Biology Research Center, Graduate School of Science, Nagoya University, Nagoya, Japan; 9 Graduate School of Science and Engineering, Tokyo Metropolitan University, Tokyo, Japan

## Abstract

Numb functions as an oncosuppressor by inhibiting Notch signaling and stabilizing p53. This latter effect depends on the interaction of Numb with Mdm2, the E3 ligase that ubiquitinates p53 and commits it to degradation. In breast cancer (BC), loss of Numb results in a reduction of p53-mediated responses including sensitivity to genotoxic drugs and maintenance of homeostasis in the stem cell compartment. In this study, we show that the Numb–Mdm2 interaction represents a fuzzy complex mediated by a short Numb sequence encompassing its alternatively spliced exon 3 (Ex3), which is necessary and sufficient to inhibit Mdm2 and prevent p53 degradation. Alterations in the Numb splicing pattern are critical in BC as shown by increased chemoresistance of tumors displaying reduced levels of Ex3-containing isoforms, an effect that could be mechanistically linked to diminished p53 levels. A reduced level of Ex3-less Numb isoforms independently predicts poor outcome in BCs harboring wild-type p53. Thus, we have uncovered an important mechanism of chemoresistance and progression in p53-competent BCs.

## Abstract (precis)

Numb regulates the activity of the tumor suppressor p53 by inhibiting Mdm2. This study from Colaluca et al. highlights the structural and molecular bases of Numb–Mdm2 interaction and shows how Numb splicing impacts specifically on p53 regulation and breast cancer prognosis.

## Introduction

By asymmetrically partitioning at mitosis in both developmental systems and stem cell (SC) compartments, Numb imparts alternative fates to daughter cells (Uemura et al., 1989; Rhyu et al., 1994; Pece et al., 2011). The function of Numb has been linked to its ability to counteract the action of the membrane signaling receptor Notch (Guo et al., 1996). Numb can also bind to Mdm2 (Juven-Gershon et al., 1998; Colaluca et al., 2008), thereby inhibiting its ubiquitin–ligase activity on p53, which physiologically destines the latter to proteasomal degradation (Honda et al., 1997; Honda and Yasuda, 2000). As a result, Numb stabilizes the levels of p53 (Colaluca et al., 2008). This mechanism is relevant to fate determination in the mammary gland. At mitosis of the mammary SC, Numb partitions preferentially into one of the daughter cells. This in turn imposes to that daughter an SC fate as a result of Numb-dependent high levels of p53, which are responsible for its withdrawal into quiescence (Tosoni et al., 2015), a hallmark of stemness (Cheung and Rando, 2013). These findings are relevant to breast cancer (BC), where there is frequent attenuation of Numb expression (Pece et al., 2004; Rennstam et al., 2010), an event that correlates with an adverse prognosis (Colaluca et al., 2008). We have shown that the control of Numb over p53 represents physiologically a tumor suppressor barrier that prevents the uncontrolled expansion of the SC compartment (Tosoni et al., 2015, 2017). Loss of Numb leads to the emergence of cancer SCs (CSCs), an effect that can be rescued by pharmacological inhibition of Mdm2 with ensuing stabilization of p53 (Tosoni et al., 2015, 2017).

These results argue that restoration of the Numb–p53 axis might represent an anti-CSC therapy in Numb-defective BCs. In this regard, it is noteworthy that different isoforms of Numb exist, which mediate distinct cellular and developmental functions (Verdi et al., 1996, 1999; Dho et al., 1999; Karaczyn et al., 2010). The most abundantly expressed isoforms differ in the presence of two alternatively spliced exons (Ex3 and Ex9; Verdi et al., 1999). Although the biological role and biochemical interactions of Ex9 have been extensively studied (Verdi et al., 1999; Dooley et al., 2003; Toriya et al., 2006; Bani-Yaghoub et al., 2007; Bechara et al., 2013; Krieger et al., 2013; Zong et al., 2014; Rajendran et al., 2016), Ex3 remains poorly characterized. In this study, we demonstrate that the sequence encoded by Ex3 (11 aa) is responsible for binding to Mdm2 and recapitulates the effects of holo-Numb on p53 and p53-dependent phenotypes. We present a detailed structural and biochemical characterization of the Numb–Mdm2 binding interface, which reveals the molecular basis of the interaction and paves the way for the design of small molecules to restore Numb function in Numb-defective BCs. Finally, we show that chemoresistance and an aggressive disease course in human BCs correlate with low expression of p53-stabilizing isoforms 1 and 2 of Numb.

## Results

### The phosphotyrosine binding (PTB) domain of Numb interacts directly with the acidic domain of Mdm2

**Figure 1.**
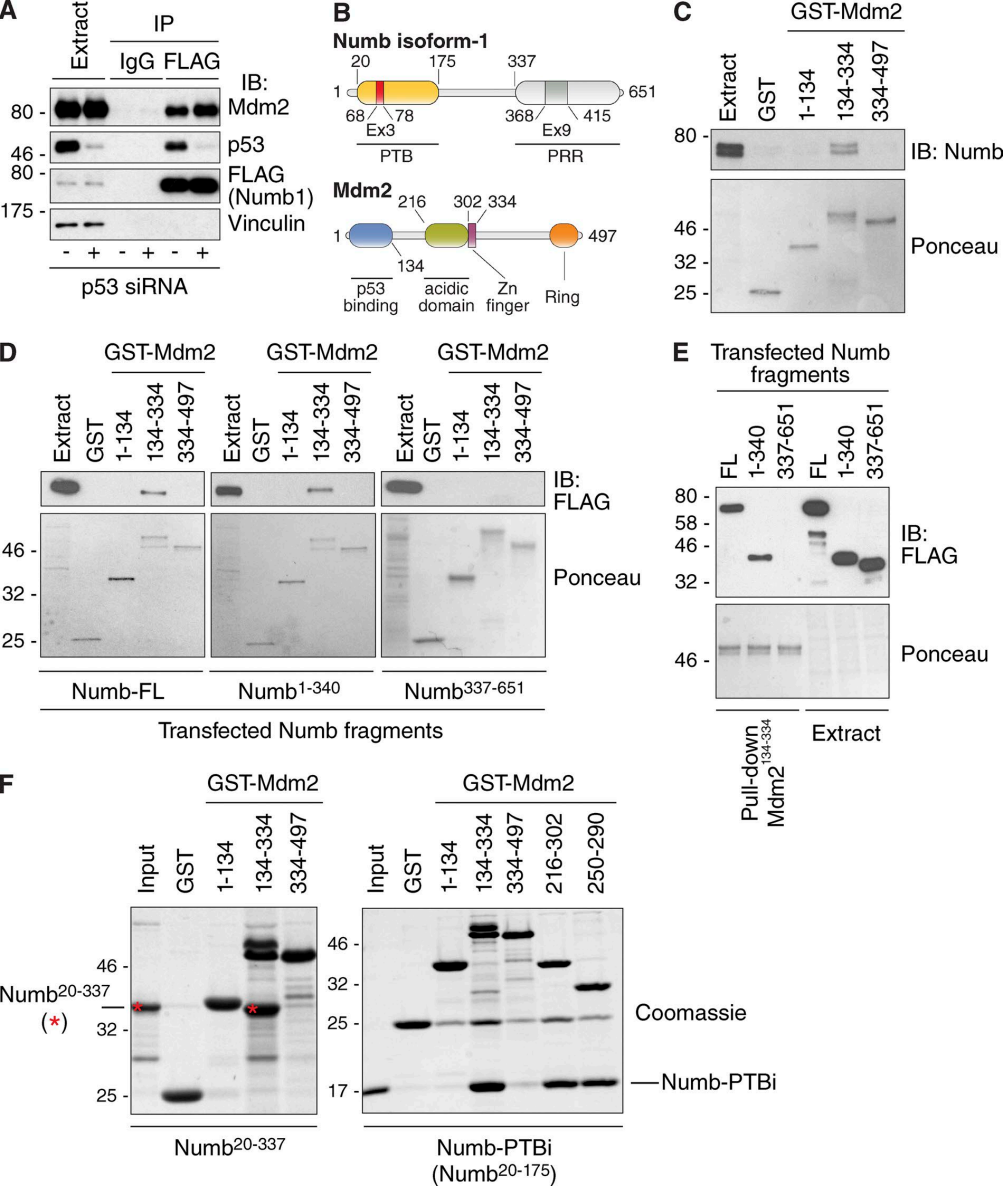
**The PTB domain of Numb interacts directly with the acidic domain of Mdm2. (A)** HEK293 cells were transfected with siRNA oligonucleotides targeting p53 (+) or control oligonucleotides (−) and after 24 h were further transfected with the catalytically inactive Mdm2-C464A mutant (to avoid p53 degradation) and FLAG–Numb-1. Immunoprecipitates with anti-FLAG or irrelevant IgG were immunoblotted as shown. Extract was 0.01% of the IP. **(B)** Domain structure of human Numb isoform-1 (Numb-1) and Mdm2. The two regions (Ex3 and Ex9) deriving from differential splicing of exon 3 and 9 are both present in Numb-1. PRR, proline-rich region. **(C)** Pulldown of endogenous Numb (from 1 mg of MCF-10A lysate) with GST-Mdm2 fragments (0.5 µM). (Top) Numb IB; (bottom) Ponceau staining. Endogenous Numb is frequently resolved as a doublet (top band, Numb-1 and -3; bottom band, Numb-2 and -4). Extract was 0.025% of the IP. **(D)** The purified GST-Mdm2 fragments shown at the top (0.4 µM) were used to pull down the Numb fragments (from Numb-1; expressed as FLAG-tagged in HEK293 cells; 0.75 mg of lysate) shown at the bottom. Top, FLAG IB; bottom, Ponceau staining. Extract was 0.025% of the IP. **(E)** Comparative pulldown of Numb fragments as in D with purified GST-Mdm2^134–334^ (numbering according to UniProtKB/Swiss-Prot: Q00987). FL, full length. **(F)** The GST-Mdm2 fragments (1 µM) were used to pull down the purified Numb fragment 20–337 and Numb PTBi (bacterially expressed as His fusions; 5 µM). Detection was done by Coomassie staining. Asterisks mark Numb^20–337^. Molecular masses are given in kilodaltons.

In the context of the Numb–Mdm2–p53 trimeric complex, binding of Numb to Mdm2 prevents ubiquitination and degradation of p53 (Colaluca et al., 2008). However, the Numb–Mdm2 association is independent of p53 (Fig. 1 A; Juven-Gershon et al., 1998; Colaluca et al., 2008). The regions responsible for the interaction were mapped by using three GST-fused fragments of Mdm2 to recover endogenous Numb or FLAG-tagged Numb fragments (derived from the longest Numb isoform, Numb-1, containing both Ex3- and Ex9-coded sequences) from cellular lysates (Fig. 1, B–E). This allowed mapping of the binding surfaces to the central domain of Mdm2 (Mdm2^134–334^, containing its acidic region) and the N-terminal PTB-containing fragment (Numb^1–340^) of Numb. The interaction is direct, as shown by assays performed with purified proteins, which further narrowed the surfaces to the PTB domain of Numb (Numb^20–175^, as present in the Ex3-containing Numb isoforms) and to a short stretch of the acidic domain of Mdm2 (positions 250–290; Fig. 1 F).

### The Ex3-encoded sequence of Numb PTB is necessary for interaction with Mdm2

**Figure 2.**
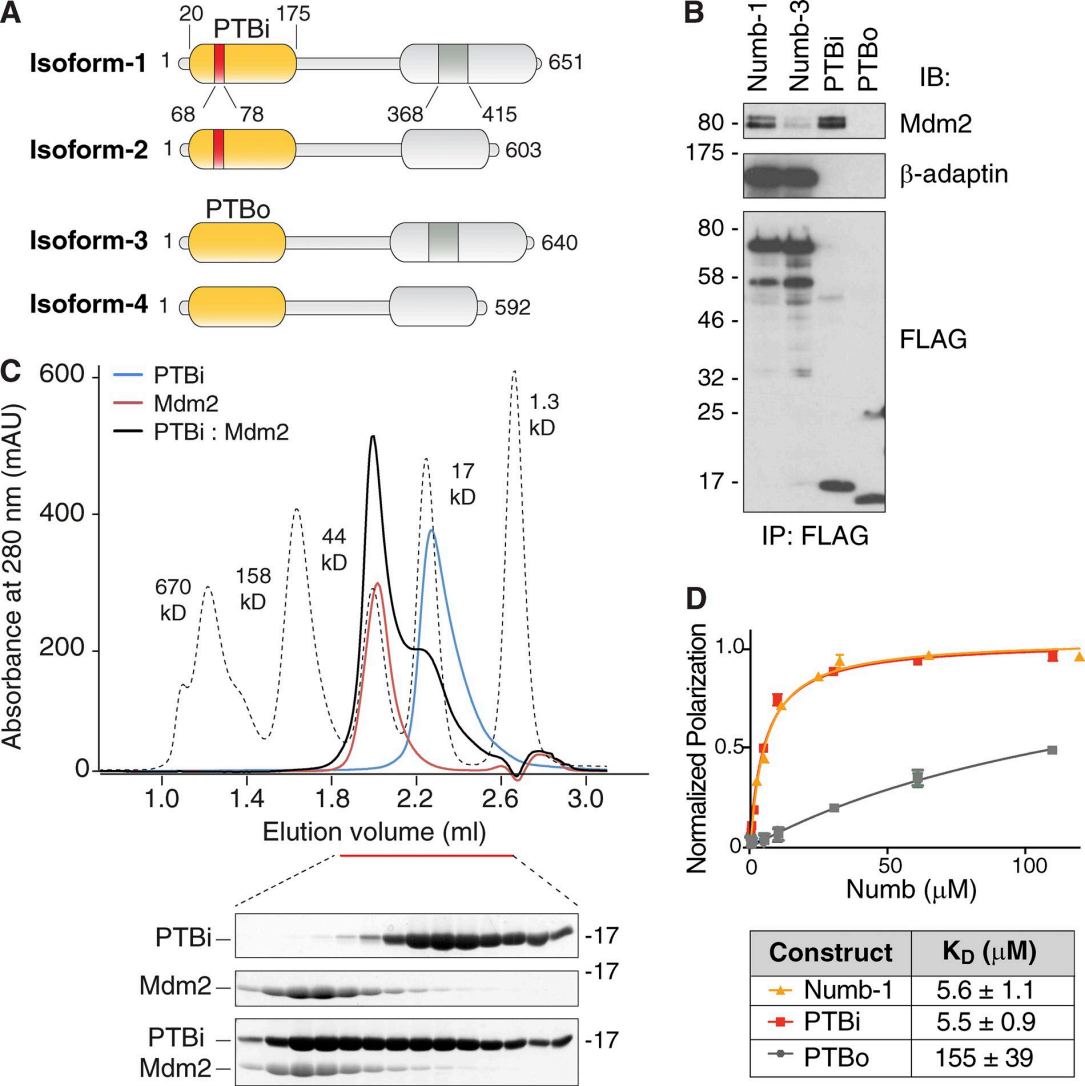
**The PTBi–Mdm2 interaction requires the Ex3-coded sequence. (A)** Schematic representations of the four human Numb isoforms. **(B)** HEK293 cells were cotransfected with Mdm2 and the indicated FLAG-tagged Numb constructs. Anti-FLAG IPs were analyzed in IB as indicated. β-Adaptin was used as a positive control for binding to full-length Numb-1/3. **(C)** SEC elution profiles of the Numb^PTBi^–Mdm2^216–302^ complex and of individual subunits with corresponding Coomassie-stained SDS-PAGEs of the peak fractions. Species were injected in a Superdex-200 column at a 300-µM concentration. Mdm2^216–302^ in isolation eluted around the 44-kD molecular weight marker as the Numb^PTBi^–Mdm2^216–302^ complex because of its unstructured conformation (see also Fig. S3, A and B). Because of the highly dynamic nature of the interaction, the Numb–Mdm2 complex partially dissociated during the SEC run. Mdm2^216–302^ stained poorly, likely as a result of its acidic composition. **(D)** FP measurements of binding affinity between rhodamine-labeled Mdm2^216–302^ and Numb isoform-1 full-length, Numb-PTBi, and Numb-PTBo. The data (*n* = 3; means ± SD) were fitted to a curve as described in the Fluorescence polarization (FP) section of Materials and methods. Molecular masses are given in kilodaltons.

In the four isoforms of Numb, the PTB domain is present as a short version (PTBo in isoform 3 and 4) or a long one (PTBi in isoform 1 and 2), differing for the presence of a stretch of 11 aa coded by Ex3 (Fig. 2 A). We investigated whether this sequence is required for binding to Mdm2. Numb-1 or its isolated PTBi expressed in cells were able to coimmunoprecipitate with Mdm2, whereas Numb-3 or its PTBo showed much reduced interaction (Fig. 2 B). Next, we performed a quantitative characterization of the interaction. For these experiments, because the minimal Mdm2 fragment (Mdm2^250–290^) was unstable at high concentrations, we used the more stable fragment Mdm2^216–302^. In size-exclusion chromatography (SEC), equimolar amounts of purified PTBi and Mdm2^216-302^ eluted in a 1:1 complex (Fig. 2 C). Finally, purified Numb-1 or the PTBi bound to Mdm2^216–302^ with similar binding affinities, whereas the PTBo displayed much higher K_D_ (Fig. 2 D).

### The Ex3-encoded sequence of Numb PTB is a novel binding surface

**Figure 3.**
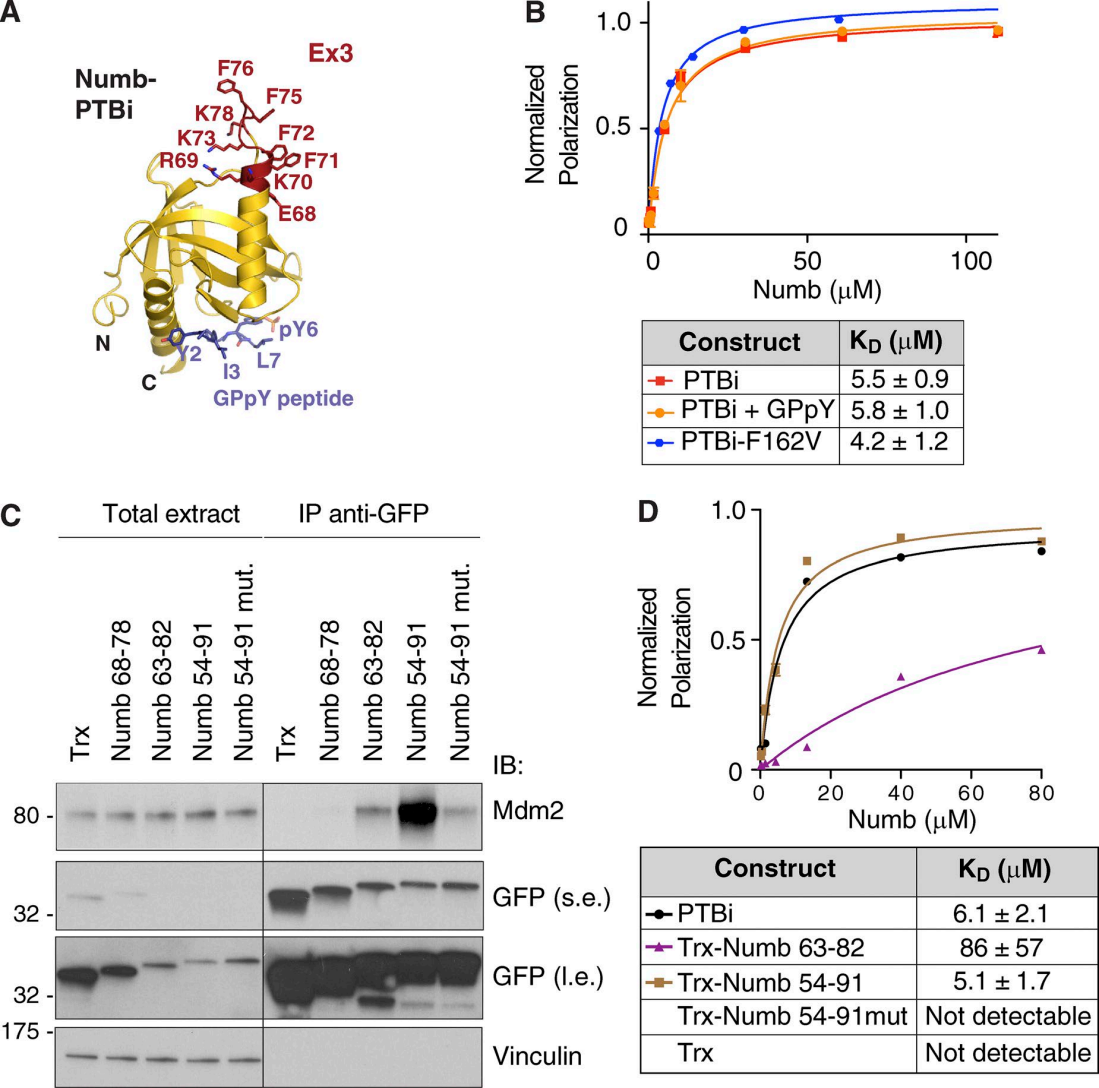
**Characterization of the minimal region of Numb binding to Mdm2. (A)** Ribbon model of Numb-PTBi in complex with the GPpY phosphopeptide. The PTBi Ex3 insert is displayed in red as balls and sticks, and the GPpY peptide is shown in blue. **(B)** FP measurements of binding affinity between rhodamine-labeled Mdm2^216–302^ and PTBi (reported for comparison; see also Fig. 2 D), PTBi in complex with GPpY (preassembled at a PTBi concentration of 200 µM with a 1.5-molar excess of peptide), and PTBi harboring the F162V mutation. As shown, the binding to Mdm2^216–302^ was not affected by the presence of GPpY or by the F162V mutation that impairs the binding of PTBi to the GPpY peptide. *n* = 4. **(C)** The thioredoxin (Trx)-based constructs (see Fig. 4 D for the amino acid sequence) harboring the fragments of the PTBi indicated at the top (all engineered as GFP fusion proteins; Trx, empty thioredoxin scaffold), were transfected in HEK293 cells together with Mdm2. Anti-GFP IPs were immunoblotted as indicated. The Numb 54–91 mutant construct harbored four mutations, in which R69, K70, K73, and K78 were replaced with glutamic acid. Total extract was 0.005% of the IP. The black line indicates that intervening lanes were spliced out. Molecular masses are given in kilodaltons. l.e., long exposure; s.e., short exposure. **(D)** FP measurements of binding affinity between rhodamine-labeled Mdm2^216–302^ and Numb-PTBi or the indicated thioredoxin-Numb fusion proteins (bacterially expressed as His fusions). The data (*n* = 3; means ± SD) were fitted to a curve as described in the Fluorescence polarization (FP) section of Materials and methods.

We determined the crystallographic structure of Numb-PTBi bound to a phosphopeptide (GPpY) derived from a “canonical” PTB-binding adapter protein (Li et al., 1998) at 2.8 Å resolution. In the two crystal forms obtained (Table S1), the canonical PTB fold was extended by the insertion of the Ex3-encoded sequence (Fig. 3 A). The 11-residue Ex3 loop was found to adopt three conformations, all characterized by extension of helix α2 of one or two turns. Ex3 contributed to the formation of a large positively charged patch contributed by lysines 63, 66, 81, 82, and 85 and arginine 64 from Ex3 and flanking regions in the core PTB fold (Figs. 3 A and S1, A–C). Besides Ex3 residues, the overall fold of all PTBi conformers was indistinguishable from that of the mouse Numb PTBo (PDB ID 3F0W), with a root mean square deviation for the Cα trace of the common residues of 0.40 Å. In addition, the “canonical” phosphopeptide-binding region of the PTB domain was remote and completely distinct from the Ex3-coded region (Figs. 3 A and S1, A–C). Indeed, by a variety of approaches, we were able to show that the peptide-binding cleft of PTBi is not involved in the interaction with Mdm2 (Figs. 3 B and S2, A–C).

These results argue that the Ex3 sequence mediates the binding of PTBi to Mdm2. We took advantage of the structure of the Numb-PTBi to engineer fragments comprising the Ex3 region into a thioredoxin scaffold. The Ex3-coded region alone (11 aa; Numb^68–78^) could not be immunoprecipitated with Mdm2 (Fig. 3 C). However, the addition of short flanking sequences from the Numb-PTBi (Numb^63–82^ and Numb^54–91^) resulted in a readily detectable interaction in vivo, which could be strongly reduced by mutating the positively charged residues of the Ex3-coded region into Glu (Numb^54–91–mut^; Fig. 3 C). Quantitative assessment of the interactions reveals a hierarchy of affinities, with Numb^54–91^ fully recapitulating the binding strength of Numb-PTBi (Fig. 3 D). We concluded that the 11-aa sequence encoded by Ex3 is required in the context of a surface that is necessary and sufficient for high-affinity Numb binding to Mdm2.

### Characterization of the PTBi–Mdm2 interaction

**Figure 4.**
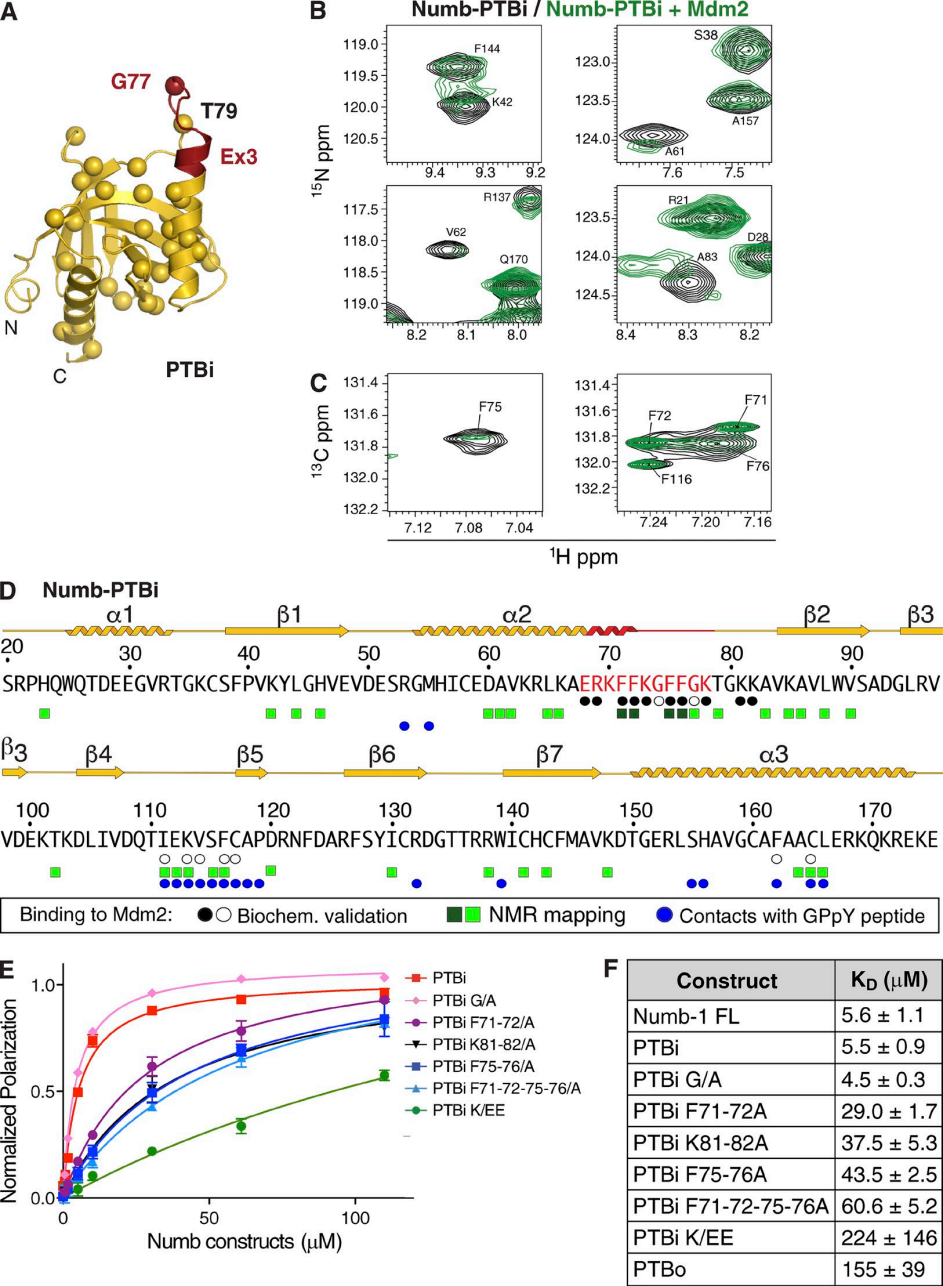
**Characterization of the PTBi–Mdm2 interface on the PTBi side. (A)** Cartoon representation of the crystal structure of PTBi colored as in Fig. 3 A. Amides of residues experiencing chemical shift changes upon Mdm2 binding are displayed as spheres colored in red for strongly affected residues in and near the Ex3 insert. **(B and C)** Selected NMR signals are shown from [^1^H][^15^N]HSQC spectra of [^15^N] uniformly labeled PTBi (B) or for [^1^H][^13^C]HSQC spectra recorded with Phe-SAIL–labeled PTBi (C), free (black) and in the presence of 1.1–1.2 equivalents of Mdm2^216–302^ (green). See also Fig. S3, C and D. **(D)** Summary of NMR spectral changes in PTBi (CSP or line broadening) upon Mdm2 binding. Residues that show either CSPs or intensity reductions are depicted for >0.15 ppm or >1 SD of mean intensity (light green squares) or >0.3 ppm or >2 SD of mean intensity (dark green squares). Residues whose replacement abrogates or does not affect binding to Mdm2^216–302^ (by FP or GST pulldown experiments) are labeled with black and white circles, respectively. Residues of the PTBi contacting the GPpY peptide in the crystallographic structures are marked with blue circles. The secondary structure of the PTBi as defined by crystallographic structures and NMR data are indicated above the sequence with the same color code as in A. **(E)** FP measurements of the binding affinity between rhodamine-labeled Mdm2^216–302^ and Numb-PTBi WT (PTBi) or mutated as indicated (the PTBi curve is from the same experiment described in Fig. 2 D). The data (*n* = 6; means ± SD) were fitted to a curve as described in the Fluorescence polarization (FP) section of Materials and methods. **(F)** Summary of FP experiments (see E and Fig. 2 D) to measure the binding affinities between rhodamine-labeled Mdm2^216–302^ and the indicated Numb species (means ± SD). FL, full length; PTBi-G/A, G74A-G77A substitutions; PTBi-K/EE, R69E-K70E-K73E-K78E substitutions.

We next investigated the molecular details of the PTBi–Mdm2 binding interface. Experimental nuclear magnetic resonance (NMR) chemical shifts and [^15^N] relaxation data demonstrate that Mdm2^216–302^ is an intrinsically unstructured and highly flexible monomeric protein in solution, as confirmed by static light scattering analysis (Fig. S3, A and B), thereby precluding crystallographic studies. Therefore, we used NMR spectroscopy to assess spectral changes upon formation of the PTBi–Mdm2 complex. First, we analyzed NMR data of specifically isotope-labeled PTBi upon addition of unlabeled Mdm2^216–302^. As NMR signals of the Ex3 sequence exhibit signal overlap and line broadening, we used site-specific [^13^C] or stereoarray isotope labeling (SAIL) Phe labeling to enable analysis of the four Phe residues in the Ex3 loop. We observed significant changes of NMR chemical shifts and/or line broadening upon addition of unlabeled Mdm2^216–302^ involving primarily residues of the Ex3 sequence or nearby (Fig. 4, A–D; and Fig. S3, C–E). This indicates that Numb-PTBi does not undergo large conformational rearrangements upon binding to Mdm2 and that residues of the Ex3 sequence are directly involved in the interaction. Indeed, reduction or loss of binding affinity for Mdm2^216–302^ of PTBi mutants in the Ex3 sequence or in the adjacent Lys81-Lys82 confirms that the insert is the major determinant of the interaction (Fig. 4, D–F).

**Figure 5.**
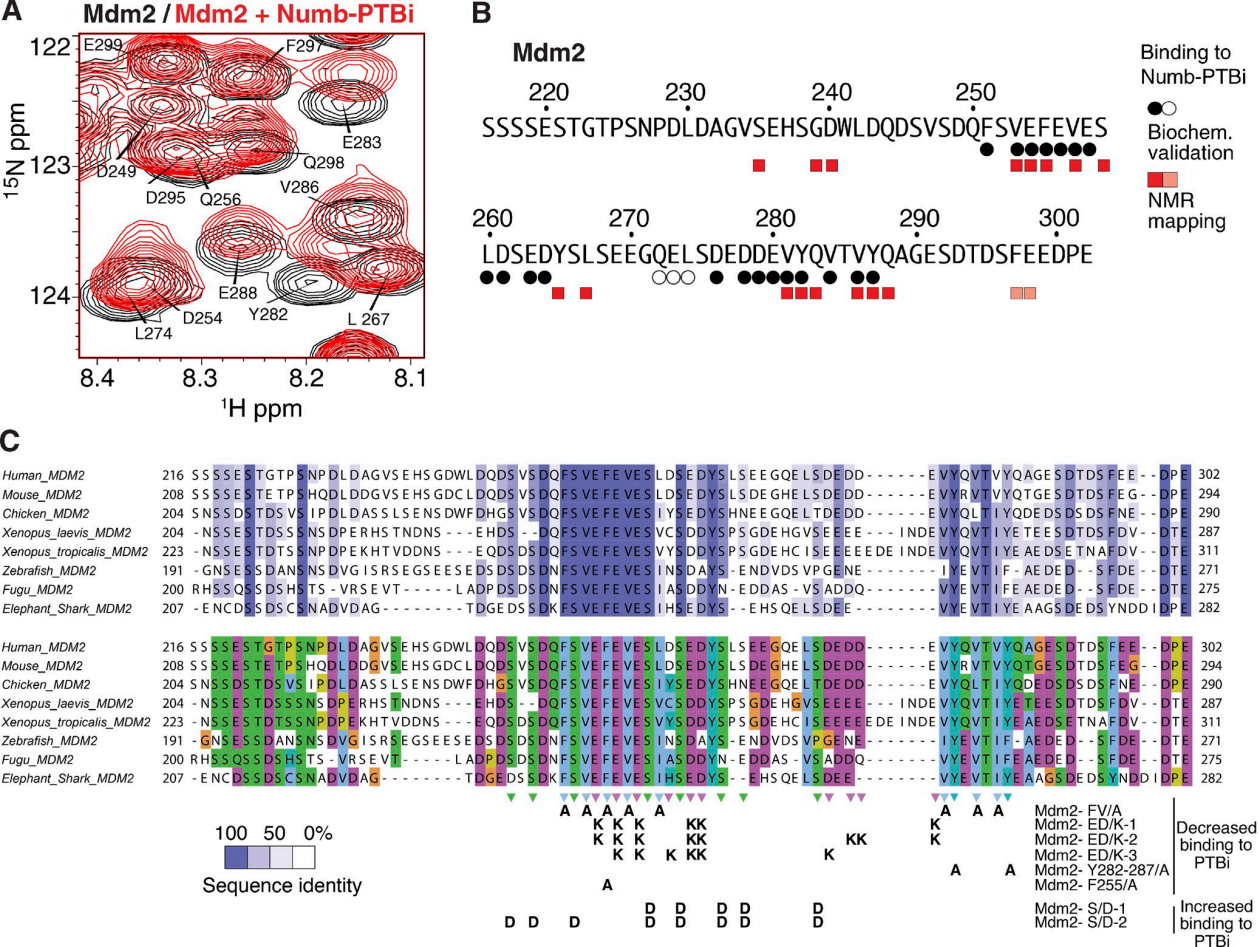
**Characterization of the PTBi–Mdm2 interface on the Mdm2 side. (A)** Region of [^1^H][^15^N]HSQC NMR spectra of [^15^N]-labeled Mdm2^216–302^ free (black) or in the presence of 1.1 equivalents of PTBi (red). **(B)** Summary of the NMR spectral changes upon binding of PTBi to Mdm2^216–302^. Residues which have a corresponding peak shift in the presence of Numb-PTBi are denoted with boxes with pink indicating 0.1–0.2 ppm and red indicating >0.2 ppm (see also Fig. S3 G). Results of the biochemical validation of Mdm2 residues at the interface with Numb-PTBi are shown in circles with the same color code as in Fig. 4 D (see also C and Fig. 6, A–F). **(C)** Summary of effects on binding to PTBi for all Mdm2 mutants tested. The mutants are shown aligned to a phylogenetic comparison among eight orthologues to evidence residue conservation and chemical nature of the residues. Top: Mdm2 residues are colored according to their conservation, which was calculated based on the alignment of eight orthologues from *Homo sapiens*, *Mus musculus*, *Gallus gallus*, *Xenopus laevis*, *Xenopus tropicalis, Danio rerio* (Zebrafish), *Takifugu rubripes* (Fugu), and *Callorhinchus milii* (Elephant shark)*.* (Middle: same alignment as above, with colors reflecting the chemical nature of the side chains. Hydrophobic side chains are in blue, negatively charged are in purple, and Ser/Thr/Gln are in green. (Bottom: summary of the Mdm2 mutants used in binding assays of Fig. 6 (A–E) to characterize the Mdm2–Numb-PTBi interface.

**Figure 6.**
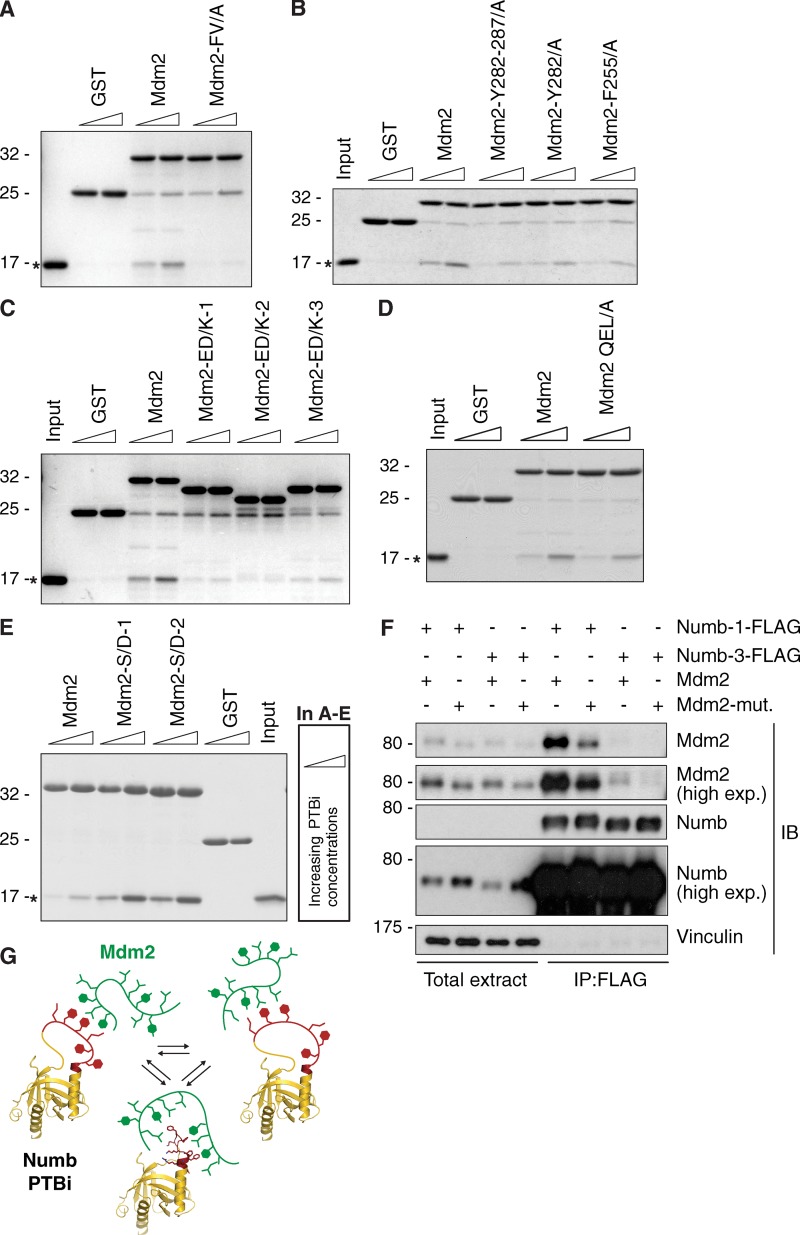
**Biochemical validation of the Mdm2 determinants required for binding to Numb-PTBi. (A–E)** Pulldown experiments with the GST proteins indicated on top (1 µM; Mdm2 mutants are graphically shown in Fig. 5 C; in Mdm2 QEL/A, the stretch 272–274 is mutated into three Ala) incubated with increasing concentrations (indicated by triangles, 2 and 4 µM, except for E, in which 1 and 2 µM were used) of PTBi (bacterially expressed and cleaved from the His moiety). Detection was by Coomassie staining. The position of the PTBi is indicated by asterisks. The other bands correspond with the GST fusion proteins. In A–D, Mdm2 mutants were engineered in the Mdm2^250–290^ background. In E, Mdm2 mutants were engineered in the Mdm2^216–302^ background so that mutagenesis of Ser246–248 could also be included. In C, the different migration of the Mdm2 mutants was probably caused by the alterations of the charges introduced by mutagenesis. **(F)** HEK293 cells were transfected with the Mdm2 constructs and FLAG-tagged Numb isoform-1 or -3 as shown on top. Mdm2-mut represents the F255A-Y282A-Y287A mutant carrying mutations in the Tyr and Phe residues important for direct interaction between the acidic domain and the PTBi (see also Fig. S3 G). Lysates were immunoprecipitated with anti-FLAG antibodies and immunoblotted as indicated. As shown, the Mdm2 mutant could not be efficiently coimmunoprecipitated with the PTBi-containing Numb-1. Numb-3 was used as a negative control for the specificity of the observed interactions. Total extract was 0.002% of the IP. Molecular masses are given in kilodaltons. **(G)** Schematic representation indicating that the PTBi–Mdm2 interaction interface represents a fuzzy complex, where two intrinsically disordered regions in both binding partners interact and retain dynamic and intrinsic disorder in the complex. Multiple negatively charged and hydrophobic motifs of Mdm2^216–302^ (green) engage the PTBi insert by charge complementarity with Lys residues and stacking interactions with phenylalanines (red).

We next mapped the PTBi binding interface onto Mdm2. NMR chemical shift perturbations (CSPs) observed for amides in Mdm2^216–302^ upon the addition of equimolar amounts of PTBi clustered around two regions containing conserved aromatic and negatively charged residues (Fig. 5, A–C; and Fig. S3, F and G). As the flexible Ex3 loop in PTBi consists of aromatic and positively charged residues, this suggests that binding of Mdm2 to PTBi is mainly driven by aromatic interactions and that they charge complementarily. Accordingly, Mdm2 variants carrying mutations in the amino acids mapped at the binding interface exhibited loss of binding to PTBi (Fig. 5, B and C; and Fig. 6, A–C), whereas no effect was observed with an Mdm2 variant carrying mutations in amino acids remote from the binding interface (Fig. 6 D). The relevance of charge complementarity for the Mdm2–PTBi interaction was further supported by the increase in binding observed using a phosphomimetic mutant of Mdm2 carrying Asp substitutions of the Ser residues in Mdm2 that are phosphorylated in vivo (Fig. 6 E; Meek and Knippschild, 2003). Finally, substitution of Mdm2 Phe255-Tyr282-Tyr287 with Ala (Fig. S3 G) was sufficient to reduce the interaction of Numb-1 with Mdm2 in cells (Fig. 6 F). We concluded that the mechanism of Numb–Mdm2 recognition is based on multiple hydrophobic and polar interactions contributed by combinations of distinct sites of the acidic domain of Mdm2 that dynamically engage the isoform-specific hydrophobic and positively charged Ex3 sequence of Numb (Fig. 6 G). Our combined data (i.e., NMR line broadening of residues in the interface) suggest that the PTBi–Mdm2 interaction represents a fuzzy complex (Miskei et al., 2017).

### The Ex3-containing region of Numb is sufficient to inhibit Mdm2

**Figure 7.**
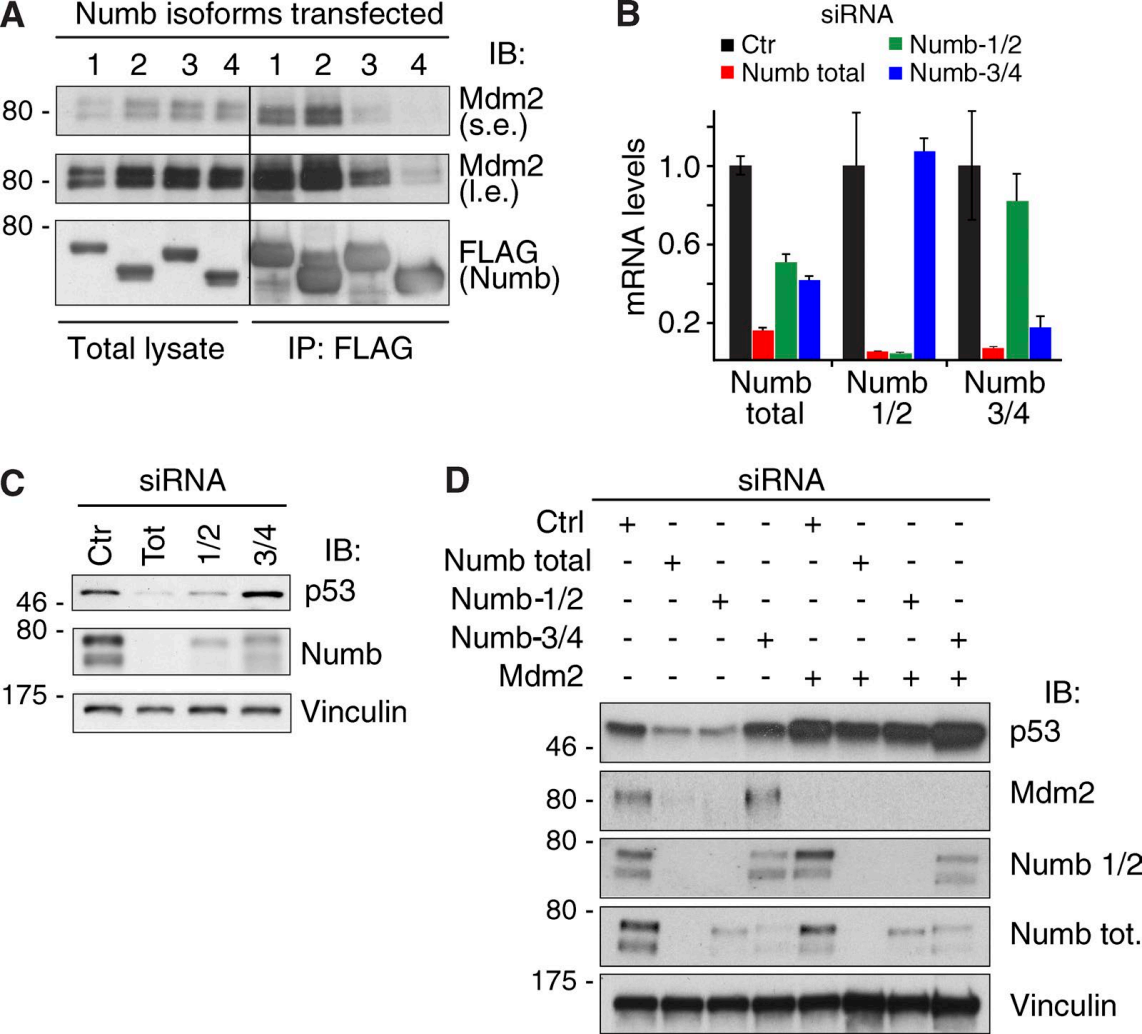
**Role of Numb isoforms in the regulation of p53 levels. (A)** HEK293 cells were transfected with the indicated FLAG-tagged Numb isoforms and Mdm2. IP (anti-FLAG) and IB were as shown. Total lysate was 0.005% of the IP. The black line indicates that intervening lanes were spliced out. **(B and C)** MCF-10A cells were silenced with siRNA specific for total Numb, Numb-1/2, or Numb-3/4 and then were analyzed by RT-qPCR (B) or IB (C). For RT-qPCR, each target was analyzed in triplicate, and the means ± SD are shown. **(D)** MCF-10A cells were silenced as indicated on the top and IB as shown on the right. Numb-1/2 is an antibody against amino acids 66–80 of human Numb isoform-1, which recognizes Numb-1 and Numb-2. Of note, Mdm2 levels are also reduced upon total Numb and Numb-1/2 silencing, making Mdm2 a transcriptional target of p53 in a negative feedback loop (Barak et al., 1993; Haupt et al., 1997; Kubbutat et al., 1997; Wu and Levine, 1997). Molecular masses are given in kilodaltons.

**Figure 8.**
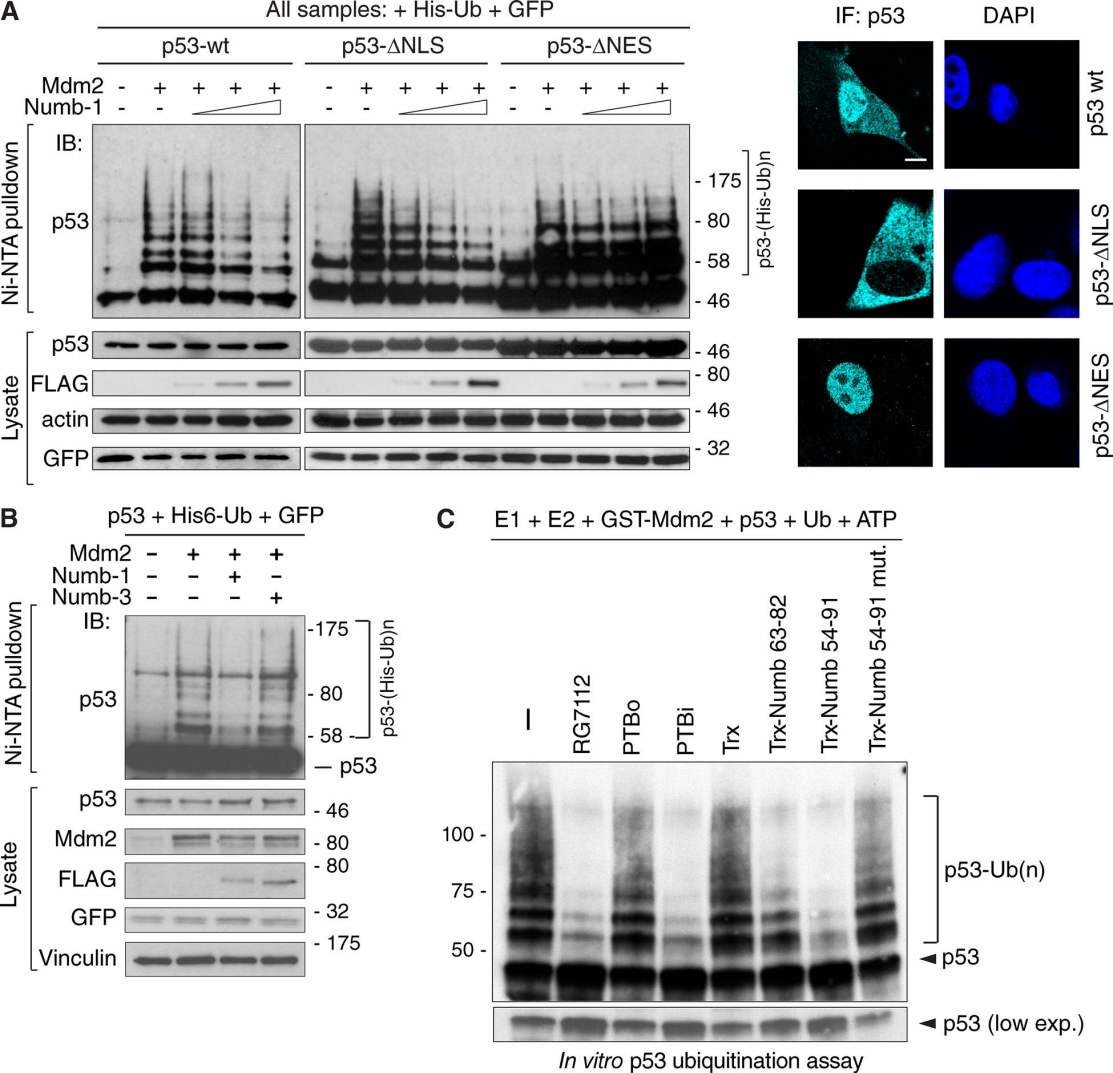
**Role of Numb isoforms in the regulation of Mdm2 activity. (A)** Numb inhibits Mdm2-dependent ubiquitination of p53 in the cytoplasm. Mdm2 and p53 shuttle between the nucleus and the cytoplasm (Roth et al., 1998; Tao and Levine, 1999), whereas the bulk of Numb is cytosolic (Santolini et al., 2000). However, p53 ubiquitination/degradation occurs both at the cytoplasmic and at the nuclear level (Joseph et al., 2003). We therefore overexpressed in H1299 cells two p53 variants with subcellular localizations restricted to the cytoplasm (p53-ΔNLS) or to the nucleus (p53-ΔNES). The p53-ΔNLS construct carries a nonfunctional p53 nuclear localization signal (with mutations of Lys305, Arg306, Lys319, Lys320, and Lys321 to Ala), and the p53-ΔNES construct carries a nonfunctional p53 nuclear export signal (with mutations of Met340, Leu344, Leu348, and Leu350 to Ala). Cells were also cotransfected with Mdm2 and Numb-1 to test the ability of the latter to inhibit in vivo the Mdm2-mediated ubiquitination of p53-WT or of its mutants. The ubiquitination pattern of the different p53 variants revealed that Numb-1 affects p53 ubiquitination only in the cytoplasm, whereas p53 ubiquitination at the nuclear level was unperturbed. Left: Mdm2-mediated p53 in vivo ubiquitination in H1299 cells transfected with the indicated plasmids and increasing concentrations of FLAG-tagged Numb isoform-1 (see also the Biochemical studies section of Materials and methods). GFP was transfected as an internal normalizer for efficiency of transfection. Lysates were subjected to IB as indicated. Right: the cellular localization of the p53 mutants was monitored by IF with anti-p53. Nuclei were counterstained with DAPI. Bar, 10 µM. **(B)** Mdm2-mediated p53 in vivo ubiquitination in H1299 cells transfected as indicated on the top (see also the Biochemical studies section of Materials and methods). GFP was transfected as an internal normalizer for efficiency of transfection. Lysates were immunoblotted as indicated. **(C)** p53 in vitro ubiquitination assay with the components and the Numb species indicated on top fused to a thioredoxin scaffold (Trx; see also the Biochemical studies section of Materials and methods). RG7112 is a small molecule belonging to the Nutlin family of Mdm2 inhibitors (designed to occupy the p53-binding pocket of Mdm2) used as control. Molecular masses are given in kilodaltons.

We reasoned that only the PTBi-containing isoforms of Numb should be able to prevent p53 ubiquitination and degradation by directly binding to Mdm2. Thus, we initially expressed the different isoforms of Numb and tested their ability to form a complex with Mdm2. Numb-1 and -2 immunoprecipitated with Mdm2 with higher efficiency than Numb-3 and -4 (Fig. 7 A). Next, we selectively ablated Numb-1/2 or Numb-3/4 (Figs. 7 B and S4 A). Depletion of all Numb isoforms or of Numb-1/2 strongly reduced p53 levels, whereas silencing of Numb-3/4 had no significant effect on p53 levels (Fig. 7 C). Importantly, the reduction in p53 levels could be rescued by the concomitant silencing of Mdm2 (Fig. 7 D). Finally, by using p53 mutants restrictively localized to the nucleus or to the cytoplasm, we showed that Numb-1 affects p53 ubiquitination only in the cytoplasm (Fig. 8 A).

We investigated whether the isoform-specific reduction of p53 levels depended on direct ubiquitination of p53 by Mdm2. In an in vivo ubiquitination assay, expression of Mdm2 and p53 in p53-null H1299 cells yielded a basal level of p53 ubiquitination, which could be reverted by concomitant expression of Numb-1 but not Numb-3 (Figs. 8 B and S4 B). Similarly, in an in vitro ubiquitination assay (containing E1, E2, GST-Mdm2, Ub, ATP, and p53), the addition of purified PTBi but not of PTBo inhibited ubiquitination of p53 (Fig. 8 C). Importantly, addition of thioredoxin-scaffolded Numb^63–82^ and Numb^54–91^ inhibited the reaction to extents comparable to their binding affinities for Mdm2, an effect that was not exerted by the binding mutant Numb^54–91–mut^ (Fig. 8 C).

### PTBi-containing isoforms of Numb sustain p53-mediated responses to chemotherapeutic agents

**Figure 9.**
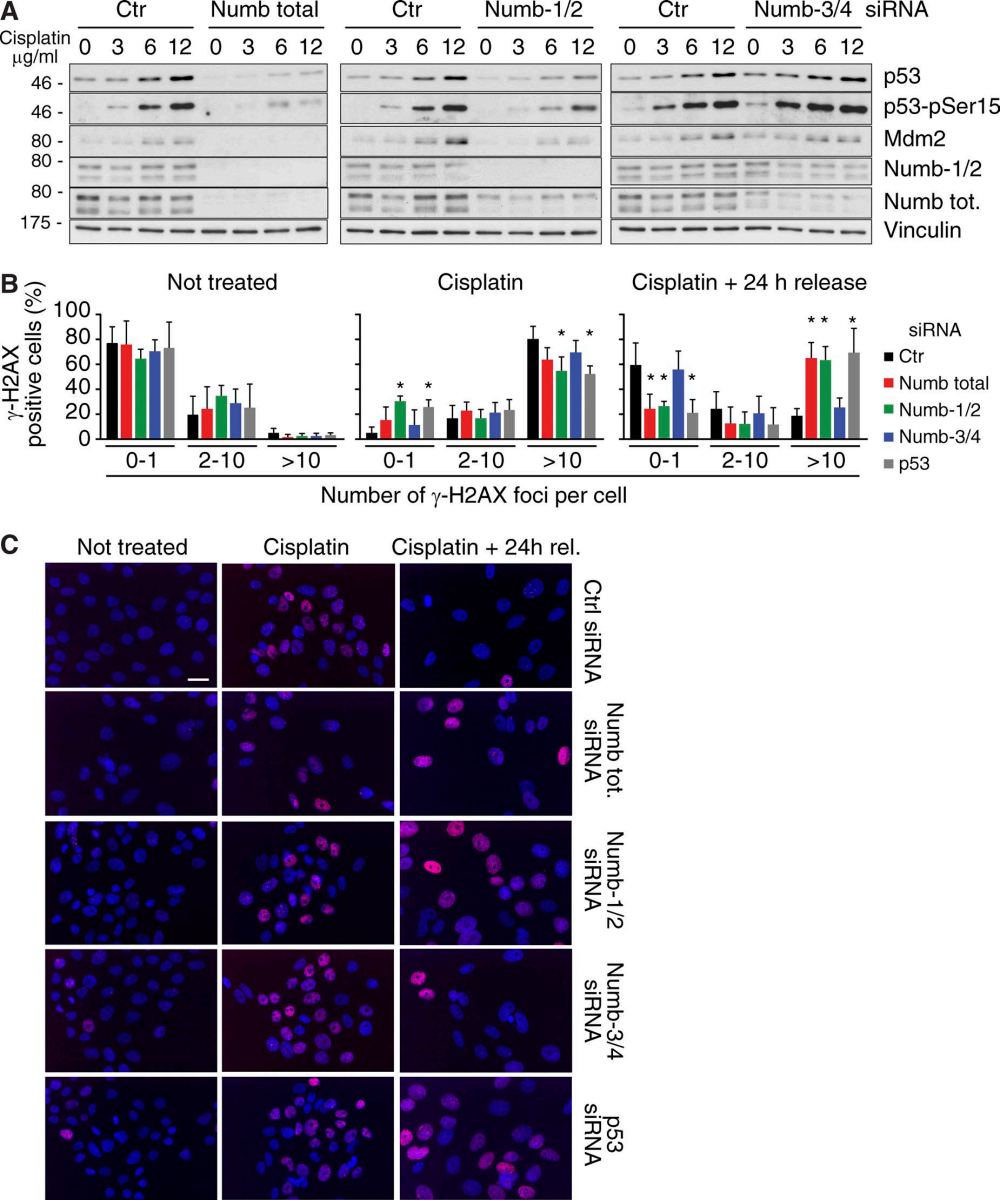
**Role of Numb isoforms in regulating the p53 response to genotoxic stress. (A)** MCF-10A cells silenced for the indicated Numb isoforms were treated with increasing doses of cisplatin for 16 h and IB as indicated. p53-pSer15 represents the form of p53 activated by phosphorylation of Ser15, detected with a specific antibody. Molecular masses are given in kilodaltons. **(B)** MCF-10A cells silenced as shown on the right were left untreated (left) or treated with 12 µg/ml cisplatin for 15 h (middle) and then released in fresh medium for 24 h (right). IF with antiphosphorylated γ-H2AX was performed. The γ-H2AX foci per cell were counted in at least five random fields for each condition (from two independent experiments). In each field, the percentage of the cells with 0–1, 2–10, and >10 foci per nucleus was evaluated, and the mean of these percentages for each condition was reported in graph with its SD. *, P ≤ 0.01 compared with control in each group. **(C)** Representative panels of the MCF-10A cells used in B (and silenced as shown on the right) stained with antiphosphorylated γ-H2AX (red) and with DAPI (blue). Bar, 50 µM.

Numb ablation results in impaired p53 levels and transcriptional activity in response to genotoxic stress in MCF-10A cells (Fig. 9 A; Colaluca et al., 2008). To test whether this function is isoform specific, we selectively silenced isoforms-1/2 versus isoforms-3/4 in MCF-10A cells. Silencing of Numb-1/2 but not Numb-3/4 significantly affected p53 levels and activation in response to cisplatin (Figs. 9 A and S4 C). Consistent with the notion that Mdm2 is a transcriptional target of p53 in a negative feedback loop (Barak et al., 1993; Haupt et al., 1997; Kubbutat et al., 1997; Wu and Levine, 1997), Mdm2 levels were also markedly reduced upon cisplatin treatment in Numb-1/2 (or in Numb total) depleted cells (Fig. 9 A).

Alterations of Numb-1/2 levels should result also in perturbations in p53-mediated responses such as DNA damage repair. The phosphorylation at serine 139 of histone H2AFX (γ-H2AX) and the number of γ-H2AX foci are considered to be a marker of DNA damage and their persistence an indication of prolonged DNA damage (Rogakou et al., 1998; Paull et al., 2000). MCF-10A cells were treated with cisplatin and then washed free of the drug and monitored for ≤24 h. In p53-knockdown (KD), total Numb-KD, and Numb-1/2–KD cells, we detected after 24 h an increased number of cells with >10 γ-H2AX foci than in control and Numb-3/4–KD cells, indicating persistence of DNA damage during the cisplatin chase (Fig. 9, B and C).

**Figure 10.**
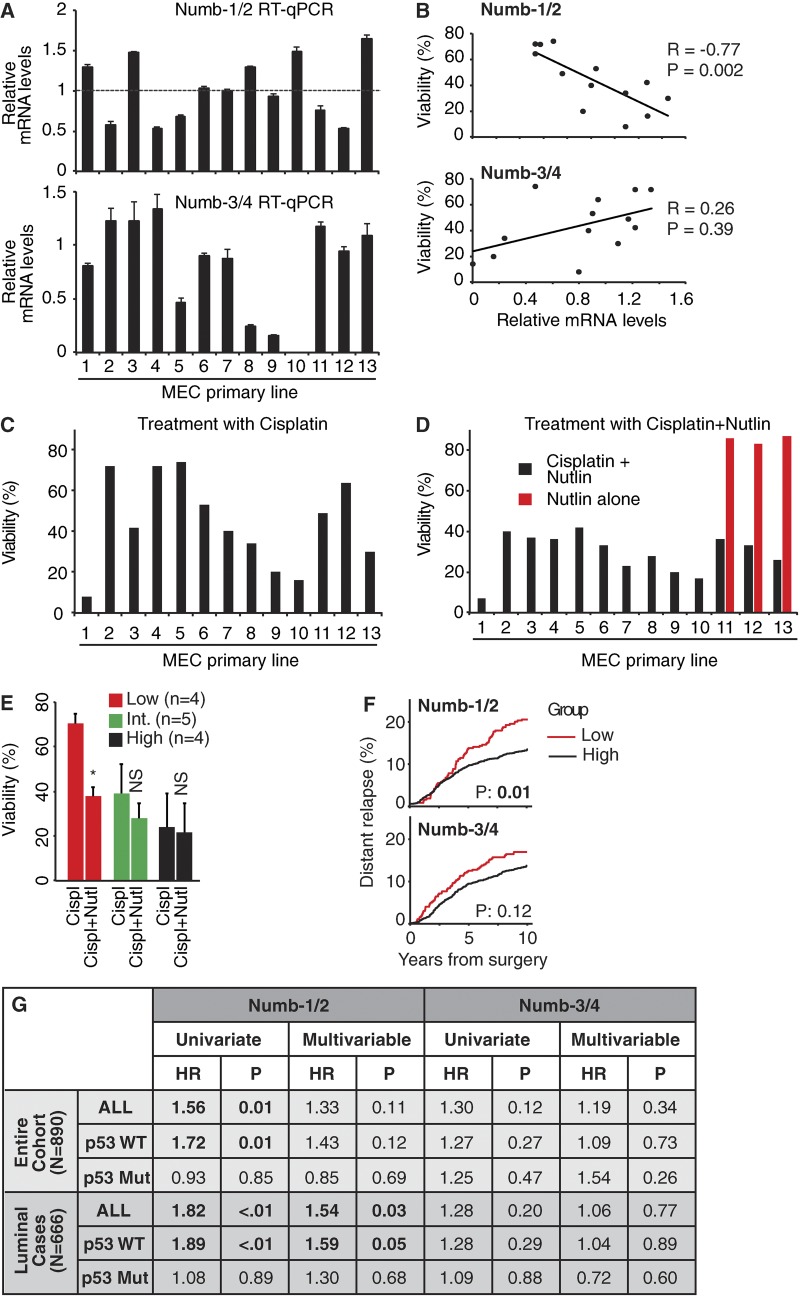
**Numb isoforms in response to genotoxic stress and prognosis of BCs. (A)** RT-qPCR analysis of the mRNA expression of Numb isoforms in tumor primary breast mammary epithelial cells (MECs) derived from 13 patients. Data are from a single experiment run in triplicate and expressed as mean ± SD relative to mRNA levels in MCF-10A cells (=1). **(B–E)** Survival of primary BC cells treated with cisplatin (18 µg/ml for 9 h) or cisplatin + Nutlin-3 (10 µM) followed by release in fresh medium for 72 h. Data are from a single experiment. In B, the viability of the cells after cisplatin treatment (plus release in fresh medium) is indicated as a function of the mRNA levels of Numb isoforms-1/2 or -3/4 shown in A (normalized to MCF-10A levels). R is the Pearson correlation coefficient calculated for the linear relationship in each graph with the relative p-value (P; two-tailed Student’s *t* test). The viability of the cells after cisplatin alone (C) or cisplatin + Nutlin-3 (plus release in fresh medium; D) is shown. As control to evaluate the effect of Nutlin-3 on cell viability, BC cells from three patients were also treated with Nutlin-3 alone (D, red bars). In E, data from C and D are shown as a bar graph (means ± SD) combining primary cells displaying high, intermediate (Int.), or low levels of Numb (defined as tertiles of expression). *, P < 0.01 compared with cisplatin alone in each group. **(F)** Cumulative incidence of distant metastasis in 890 BC patients clustered in quintiles according to the mRNA level of the indicated Numb species. Red, low Numb isoforms (lowest quintile; lowest 20%; *n* = 178); black, high Numb isoforms (other four quintiles combined; highest 80%; *n* = 712). P-values from a univariate Cox proportional hazard ratio are shown. See also the RNA purification and quantitative real-time PCR analysis and the Selection of breast cancer patients and statistical analysis sections of Materials and methods for details. **(G)** Hazard ratio (HR) of distant metastasis (with corresponding p-values) of low versus high Numb (defined as in F) in the entire case cohort (*n* = 890) or in the luminal subtype of BC (*n* = 666), further stratified for p53 mutational status (see also Fig. S4, E and F). Univariate and multivariable analyses are shown with corresponding p-values (see the Selection of breast cancer patients and statistical analysis section of Materials and methods). In the entire case cohort (*n* = 890), multivariable analyses were adjusted for grade, Ki-67, tumor size, number of positive lymph nodes, age at surgery, ERBB2 status, and estrogen/progesterone receptor status, whereas in the luminal subtype of BC (*n* = 666), multivariable analyses were adjusted for grade, Ki-67, tumor size, number of positive lymph nodes, and age at surgery.

These results demonstrate that the presence of the 11-aa insert encoded by Ex3 in the PTBi of Numb sustains p53-mediated responses to genotoxic stress. This predicts that BCs with reduced levels of isoform-1/2 should display resistance to genotoxic agents. To address this issue, we used 13 primary lines of mammary epithelial cells from BC patients (all lines harbored WT p53). In these cells, there was a significant inverse correlation between the mRNA levels of Numb-1/2 and cisplatin sensitivity, whereas no significant correlation could be evidenced with Numb-3/4 (Fig. 10, A–C). To assess whether the scarce effect of cisplatin in Numb-1/2^LOW^ cells was caused by a lack of p53-mediated responses, we restored the levels of p53 with the Mdm2 inhibitor Nutlin-3 (Vassilev et al., 2004). This restored the sensitivity to cisplatin in Numb-1/2^LOW^ cells (Fig. 10, D and E).

### Numb isoforms predict an aggressive disease course in human BCs

We have previously shown that loss of Numb expression is relevant to BCs and predicts a poor prognosis (Colaluca et al., 2008). We reasoned that BCs displaying reduced levels of Numb-1/2, being resistant to genotoxic agents, might also display poorer disease outcome. Thus, we assessed the relevance of the Numb isoforms, measured by reverse transcription–quantitative PCR (qPCR; RT-qPCR), to the natural history of BCs in a case cohort of 890 patients (See also the RNA purification and quantitative real-time PCR analysis and the Selection of breast cancer patients and statistical analysis sections of Materials and methods for details). Patients were stratified as “LOW” (lowest quintile of expression) or “HIGH” (other four quintiles together; Fig. S4 D). By survival analysis, a Numb-1/2^LOW^ status was predictive of a higher risk of metastasis in univariate analysis (Fig. 10, F and G). The correlation was maintained in the p53 WT subgroup of patients, whereas it disappeared in the mutated p53 subgroup, arguing that also in real cancers the tumor suppressor function of Numb-1/2 is exerted through p53 (Figs. 10 G and S4, E and F). The significance in the entire cohort was lost in multivariable analysis (Fig. 10 G). However, when the luminal subtype of cancers was considered, which accounts for ∼70% of all BCs and is enriched in p53 WT tumors (Dumay et al., 2013), a low Numb-1/2 status emerged as an independent predictor of the risk of metastasis in multivariable analysis both in the entire luminal subcohort and in the p53-WT sub-subcohort (Fig. 10 G). Conversely, the levels of expression of Numb-3/4 did not correlate with disease outcome in any of the groups (Fig. 10, F and G). These results were not correlated with the type of adjuvant treatment, as witnessed by the lack of statistically significant differences in the chemotherapy and/or hormonal therapy received by the patients of the different groups (Table S2).

## Discussion

In this study, we show that a short 11-aa sequence contained in the PTB domain of Numb and encoded by the alternatively spliced Ex3 of the *NUMB* gene is responsible for the interaction with Mdm2 and for the inhibition of its activity on p53, with ensuing effects on the in vivo regulation of p53-dependent functions, such as response to genotoxic stress and DNA repair. With all due caution in the interpretation of data obtained with purified fragments rather than with the full-length protein, our results strongly argue that the Ex3-encoded sequence is the major structural element in the Numb moiety for binding to and inhibition of Mdm2. In support of this contention: (A) our NMR data demonstrate that the Numb–Mdm2 binding interface comprises the Ex3 sequence of PTBi and residues 216–302 of the Mdm2 acidic domain, (B) NMR data demonstrate that Ex3 in the free PTBi is intrinsically disordered and that the interaction with the intrinsically disordered region in Mdm2 remains highly flexible, (C) the binding to Mdm2 could be recapitulated by the insertion into a foreign scaffold (thioredoxin) of the 11-aa sequence flanked by minimal regions of the Numb-PTBi, which might contribute to the binding or to the proper presentation of the binding region itself, and (D) the same thioredoxin-based constructs could inhibit the Mdm2 ligase activity as efficiently as the entire PTBi.

We note that the interaction surfaces in the Numb–Mdm2 complex described in previously published papers (Juven-Gershon et al., 1998; Sczaniecka et al., 2012) are different from the ones described in our studies, in particular: (A) Juven-Gershon et al. (1998) mapped the binding surface on Numb to two regions, one contained in the C-terminal half of the PTB domain (aa 92–165 in their numbering, corresponding with aa 103–176 in our numbering for Numb isoform-1) and the other contained in the C-terminal region of the protein (aa 352–592, corresponding with aa 363–651 in our numbering for Numb isoform-1). Our results are not necessarily in disagreement with those of Juven-Gershon et al. (1998). In that paper, in fact, the reference sequence used for Numb (and accordingly, the used constructs) was that corresponding with isoform-4, which is devoid of the 11-aa insert encoded by Ex3. Our results, although clearly identifying the 11-aa insert as the major determinant of the interaction, do not exclude other lower-affinity interactions. For instance, the PTBo still displays some residual binding affinity, albeit almost 30-fold reduced with respect to the PTBi (Fig. 2 D), and Numb-3 could be coimmunoprecipitated with Mdm2, albeit at a reduced rate with respect to Numb-1 (Fig. 2 B). Interestingly, under conditions of coimmunoprecipitation, we could not detect any binding of the isolated PTBo (whereas the PTBi readily coimmunoprecipitated), further arguing for additional weak surfaces of interaction outside the PTB, possibly coinciding with the Numb^352–592^ surface mapped by Juven-Gershon et al. (1998).

Juven-Gershon et al. (1998) also mapped the sequence on Mdm2 to the intact N-terminal domain (aa 1–134) of the protein, whereas our mapping clearly identified the central acidic domain of Mdm2 as responsible for the high-affinity interaction. Again, this is not surprising because the Numb surface that they used was devoid of the Ex3-encoded sequence. Indeed, when we repeated experiments similar to those reported in Fig. 1 C with higher concentrations of Mdm2^1–134^, we also evidenced some weak positivity in the pulldown experiment (not depicted).

(B) Sczaniecka et al. (2012), by performing a peptide-based scanning of the entire PTB domain including the Ex3-derived sequence, identified the binding region of Numb in two short peptides within the PTB region (peptides “8” and “9” covering residues 113–132 and 129–148, respectively). These two peptides belong to the central β sheet of the PTB hydrophobic core and lie away from Ex3-containing region that, based on our studies, interacts with Mdm2. Thus, our mapping is at variance with that of Sczaniecka et al. (2012). To this regard, we note that:

(a) From our present data, only the isoforms of Numb containing the Ex3-coded sequence bind to Mdm2 with high affinity (Figs. 2 B and 7 A). Conversely, the prediction according to Sczaniecka et al. (2012) is that all Numb isoforms should bind comparably to Mdm2. In addition, we showed that the difference in the K_D_ between Numb PTBi or PTBo is ∼30-fold (Fig. 2 D), with a binding affinity of Numb PTBo for Mdm2 in the low millimolar range, a value that argues against significant interaction in vivo.

(b) According to our PTBi structure, the 113–148 region is an unlikely candidate for binding to Mdm2 because it belongs to the central β sheet of the PTB domain and is mostly buried in the hydrophobic core of the domain. Only a few residues of peptide 9 face the “canonical” binding groove of Numb, which, however, is not involved in binding to Mdm2 (Figs. 3 B and S2, A–C).

(c) The region of Numb spanning residues 54–91 (encompassing Ex3) and not overlapping with peptides 8–9 of Sczaniecka et al. (2012), when engineered into an unrelated scaffold, was able to recapitulate the binding properties of PTBi-containing Numb isoforms and to inhibit the E3 activity of Mdm2 (Fig. 3, C and D; and Fig. 8 C).

The combined analysis of crystallographic and NMR data revealed interesting features of the Numb–Mdm2 interaction. The crystal structure of Numb-PTBi showed that the Ex3 sequence connects the α2 helix to the β2 strand of the PTBi module adopting several conformations without altering the hydrophobic core of the domain. NMR data revealed that Ex3 is highly dynamic in solution. Most notably, the binding interface of the Numb–Mdm2 interaction is flexible and is constituted by a dynamic equilibrium of intrinsically disordered states of the Numb-PTBi-Ex3 sequence that contacts the unstructured acidic domain of Mdm2 via hydrophobic and polar interactions. Thus, the Numb–Mdm2 interaction represents a fuzzy complex (Miskei et al., 2017).

The alternative splicing of Numb is the subject of increasing interest because Numb is involved in major types of human cancers, including breast and lung cancer (Pece et al., 2004; Colaluca et al., 2008; Westhoff et al., 2009; Rennstam et al., 2010; Sheng et al., 2016; Tosoni et al., 2017). The experimental attention has been so far almost exclusively directed to Ex9 according to the observation that its splicing is developmentally regulated (Dho et al., 1999; Verdi et al., 1999; Dooley et al., 2003; Yoshida et al., 2003; Revil et al., 2010; Kim et al., 2013) and that Ex9-including isoforms promote proliferation, as opposed to the differentiation-promoting effects of Ex9-excluded isoforms (Verdi et al., 1999; Dooley et al., 2003; Toriya et al., 2006; Bani-Yaghoub et al., 2007). Mechanistic studies have hence defined several splicing-regulating factors variously regulating inclusion/exclusion of Ex9 (Bechara et al., 2013; Zong et al., 2014; Rajendran et al., 2016; Tarn et al., 2016). Alterations of these factors (for instance, RBM6, RBM10, and QKI) and of the Numb splicing machinery have been reported in human tumors (Misquitta-Ali et al., 2011; Bechara et al., 2013; Zhang et al., 2014; Zong et al., 2014), in particular in nonsmall cell lung carcinoma (NSCLC). A picture emerged suggesting that alterations of the Numb splicing machinery in NSCLC leads to preponderance of Ex9-including isoforms, which in turn can induce cell proliferation by activating the Notch pathway (Misquitta-Ali et al., 2011). How this can happen is not immediately clear considering that Numb—at least in developmental systems—is clearly an inhibitor of Notch activity (Uemura et al., 1989; Rhyu et al., 1994; French et al., 2002; McGill and McGlade, 2003). One possibility is that Ex9-containing isoforms of Numb exert a dominant-negative effect on a Notch-extinguishing function exerted by other isoforms—an issue that warrants further investigation.

Conversely, it has been reported that the levels of Ex3-containing isoforms do not change significantly in NSCLC (Misquitta-Ali et al., 2011). This is not surprising, given our present results. NSCLCs are by and large p53 mutated (Takahashi et al., 1989), and therefore, there would be no immediately obvious advantage deriving from alterations of the upstream mechanisms of regulation. The situation could be rather different in BC, where p53 is relatively less frequently mutated compared with other types of tumors (Gasco et al., 2002; Bertheau et al., 2013; Dumay et al., 2013), in particular in luminal subtypes, and loss of WT p53 activity may depend on alterations of regulatory pathways of p53 stability (Dumay et al., 2013). Indeed, we show in this study that in BCs, reduced levels of Ex3-containing Numb transcripts correlate with a worse prognosis. The fact that the prognostic power of Numb-1/2 is directly traceable to the regulation of p53 is supported by the fact that: (A) when the cohort was broken into p53-WT and p53-mutated tumors, the significance was maintained in the p53-WT subpopulation and lost in the p53-mutated one, and (B) the strongest predictive prognostic value of Numb-1/2 was observed in the subcohort of luminal BCs.

Reduced expression of Numb-1/2 might affect disease course in several ways. One possibility directly tested in this study is that it might influence the response to chemotherapy. Indeed, in a panel of primary BC lines, we could readily unmask reduced sensitivity to genotoxic agents in the Numb-1/2^LOW^ subgroup, which could be rescued by Nutlin treatment, thus directly linking this phenotype to p53 downmodulation. Another not mutually exclusive possibility is that the impact of the different Numb isoforms in tumorigenesis might reflect their differential role in developmental programs, in particular as concerns their role in balancing proliferation versus differentiation. In this contention, our recent findings that the Numb–p53 axis maintains the tumor-suppressive barrier of asymmetric division in normal mammary SCs (Tosoni et al., 2015, 2017) provides a reasonable framework to test the possibility that the decreased levels of Numb-1/2 in some BCs is causal to the emergence of CSCs. Regardless, by highlighting the molecular features of the Numb–Mdm2 interaction, our results provide a high-resolution view of how the subversion of the Numb-p53 circuitry participates to the breast tumorigenesis. The sum of our findings also argues for the possibility to exploit the knowledge of the molecular bases of Numb–Mdm2 interaction for the rational design of molecules that, by mimicking the Ex3-encoded surface of Numb, might directly inhibit Mdm2 activity to relieve p53 dysfunction in Numb-defective BCs.

## Materials and methods

### Cells and reagents

Cultivation of primary tumor human mammary epithelial cells was as described previously (Pece et al., 2004). In brief, tumor biopsies were mechanically dissociated and enzymatically digested in DMEM/F12 medium supplemented with 1 mM glutamine, 200 U/ml collagenase (Sigma-Aldrich), and 100 U/ml hyaluronidase (Sigma-Aldrich) at 37°C for 4–5 h under rotating conditions. Single-cell suspensions were obtained through an additional incubation of digested tissues in 0.05% trypsin–EDTA for 5 min at 37°C.

For survival assays (Fig. 10, B–E), primary tumor cells were plated in six-well plates. Subconfluent cells were treated with cisplatin (18 µg/ml) for 9 h and then grown in cisplatin-free medium (with or without Nutlin-3) for 72 h. Cells were then stained with 0.05% crystal violet for 10 min and then extensively rinsed with water. The crystal violet retained by live cells was leached in acetic acid (10%), and absorbance was read at 595 nm.

Antibodies were anti-Flag M2-agarose affinity gel from Sigma-Aldrich, anti-Mdm2 (OP46) from EMD Millipore, anti-p53 (DO-1 and FL-393) from Santa Cruz Biotechnology, Inc., antiphospho-p53–Ser 15 and anti-FLAG from Cell Signaling Technology, Inc., antiphosphohistone H2AX (Ser 139) from Upstate, anti-Numb monoclonal antibody (Ab21 generated in-house against amino acids 537–551 of human Numb and used to detect all isoforms of Numb) and anti–Numb-PTBi polyclonal antibody (Numb-1/2 in Figs. 7 D and 9 A generated in-house against amino acids 66–80 of human Numb isoform-1 and used to detect Numb-1 and -2), and fluorochrome-conjugated secondary antibodies were from Jackson ImmunoResearch Laboratories, Inc. Nutlin-3 (Cayman Chemical) was used at 10 µM.

### Engineering of vectors and siRNA experiments

Mammalian expression vectors for p53 and Mdm2 were a gift from K. Helin (Biotech Research and Innovation Centre, Copenhagen, Denmark). Mammalian expression vectors encoding various isoforms of Numb (or fragments) were engineered in a pcDNA vector in frame with a FLAG tag at the C terminus. GST-Mdm2 constructs for bacterial expression were cloned into a derived pGEX-6P1 vector (GE Healthcare). Numb constructs for bacterial expression were cloned into pET vectors (Novagen) in frame with a hexahistidine tag (cleavable when required by PreScission protease [Cogentech]).

The expression vector pLPC GFP-thioredoxin was a gift from G. Del Sal (Laboratorio Nazionale del Consorzio Interuniversitario per le Biotecnologie, Trieste, Italy). This vector was used to clone the Numb fragments (described in Fig. 3 C) into the thioredoxin-active site loop in the unique RsrII restriction site. Additionally, the Numb-thioredoxin inserts excised from the previous vectors were subcloned into the NdeI–XhoI restriction sites of a pET-30 vector (Novagen) in frame with a hexahistidine tag at the C terminus to generate the bacterial expression vectors used for the production of the recombinant proteins used in Figs. 3 D and 8 C.

Point mutations in various constructs were generated by QuikChange strategy (Agilent Technologies) according to the manufacturer’s instructions. The gene modifications and inserts were confirmed by DNA sequencing and/or restriction nuclease digestion.

Specific siRNAs for total Numb and the corresponding control were described previously (Pece et al., 2004). p53 siRNA (VHS40367) was from Invitrogen. For the other silencing experiments, the following siRNA oligonucleotides (GE Healthcare) were used: Numb isoforms 1 and 2, 5′-AGGCUUCUUUGGAAAAACU-3′; Numb isoforms 3 and 4, 5′-AGAUUGAAAGCUACUGGAA-3′; and Mdm2, 5′-GCCACAAAUCUGAUAGUAU-3′. Cells were transfected using Lipofectamine RNAiMAX (Invitrogen) for 72 h (final siRNA concentration, 10 nM).

### p53 gene sequencing

For p53 gene sequencing of primary BCs (Fig. 10, A–E), the p53 gene (NCBI reference sequence NM_000546) was amplified using specific primers that cover the coding sequence from the start to the stop codon (10 exons). The primer pairs were designed in intronic regions to include ≥20 nt of the splicing junctions. Every forward and reverse primer had a 5′ universal tail, a PE-21, and an M13rev sequence, respectively. This strategy allowed sequencing of all the different PCR fragments with only two sequencing primers. The primers were designed with a similar Tm so that all the different regions could be amplified simultaneously in isothermal conditions. The PCR products were purified with EXO-SAP enzymes, sequenced with BigDye chemistry (v3.1; Thermo Fisher Scientific), and run onto a 3730xl sequencer (Applied Biosystems). Data were analyzed with Mutation Surveyor (Softgenetics).

### RNA purification and quantitative real-time PCR analysis

For the screening of the case cohort (Fig. 10, F and G), total RNA was extracted from formalin-fixed paraffin-embedded (FFPE) tissue sections using the AllPrep DNA/RNA FFPE kit automated on QIAcube following the manufacturer’s instructions (QIAGEN). 200 ng of purified total RNA (measured using a NanoDrop ND-1000 spectrophotometer; Thermo Fisher Scientific) were used for first-strand cDNA synthesis using random primers and the Superscript VILO cDNA Synthesis kit (Thermo Fisher Scientific) in a final volume of 10 µl. cDNA was preamplified using a pooled TaqMan gene expression assay mix with the PreAMP Master Mix kit (Thermo Fisher Scientific) for 10 cycles following the manufacturer’s instructions and then diluted 1:5 before PCR analysis (5 µl was then used per PCR reaction).

For the extraction of total RNA (and genomic DNA for p53 sequencing) from cells (Fig. 10, A and B), the AllPrep DNA/RNA Mini kit automated on QIAcube was used following the manufacturer’s instructions (QIAGEN). 500 ng of purified total RNA were used for first-strand cDNA synthesis as previously described in a final volume of 20 µl.

qPCR was performed with hydrolysis probes (Thermo Fisher Scientific) using the TaqMan Universal PCR Master Mix (Thermo Fisher Scientific) in 10 µl of final volume in 384-well plates. Each target was analyzed in triplicate using the LightCycler 480 Real-Time PCR instruments (Roche) with the following thermal cycle conditions: 1 cycle at 95°C for 10 min followed by 45 cycles at 95°C for 15 s and 55°C for 1 min, and then 1 cycle at 40°C for 30 s.

Inventoried TaqMan gene expression assays with short amplicon sizes were: Hs01105433_m1 for “NUMB TOT” (*NUMB* isoform-1, NM_001005743; -2, NM_001005744; -3, NM_003744; and -4, NM_001005745), Hs02800695_m1 (*HPRT1*), Hs03929097_g1 (*GAPDH*), Hs99999908_m1 (*GUSB*), Hs01060665_g1 (*ACTB*), Hs00963534_m1 (*ALAS1*), and Hs00427621_m1 (*TBP*) and were all purchased from Thermo Fisher Scientific. The custom TaqMan gene expression assay to detect NUMB-1/2 (forward, 5′-GATGAATCAAGAGGAATGCACATC-3′; reverse, 5′-TGAAGAACTTCCTTTCAGCTTTCAA-3′; and probe, 5′-TGAAGATGCTGTAAAAAG-3′) was designed using the Primer Express Software V3.0 and purchased from Thermo Fisher Scientific. The custom TaqMan assay to detect NUMB-3/4 (forward, 5′-GAATGCACATCTGTGAAG-3′; reverse, 5′-ACTGCTTTAACTGCTTTC-3′; and probe, 5′-TTCCAGTAGCTTTCAATCTTTT-3′) was from Bio-Rad Laboratories.

For quantification of gene expression changes in the primary tumor cells (Fig. 10 A), the ΔΔCt method was used to calculate relative fold changes normalized against five reference genes (*GUSB*, *HPRT1*, *TBP*, *ALAS1*, and *ACTB*).

For the analysis of the RT-qPCR data from the BC case cohort (Fig. 10, F and G), each target was assayed in triplicate, and mean Cq (AVG Cq) values were calculated either from triplicate values when the SD was <0.4 or from the best duplicate values when the SD was ≥0.4. Data (mean Cq) were normalized using four reference genes (*HPRT1*, *GAPDH*, *GUSB*, and *TBP*) to account for variation in the expression of single reference genes and in RNA integrity caused by tissue fixation. The normalized Cq (Cq_normalized_) of each target gene was calculated using the following formula: Cq_normalized_ = mean Cq − SF, where SF is the difference between the mean Cq value of reference genes for each patient and a constant reference value K, and K represents the mean of the mean Cq of the four reference genes calculated across all samples (K = 24.148). This normalization strategy allowed the retention of information about the abundance of the original transcript as measured by PCR (i.e., in Cq scale), which is conversely lost when using the more classical ΔCq method. We defined Cq = 35 as the limit of detection, and Cq values beyond this limit were set to 35. Normalized data were then processed for statistical analysis.

### Selection of BC patients and statistical analysis

Analyses on BC patients presented in Fig. 10 (F and G) were performed on a case cohort of 890 patients. The case cohort was built as follows: (A) We started from a consecutive cohort of 2,453 BC patients who underwent surgery at the European Institute of Oncology (IEO) between the years 1997 and 2000 (the “IEO cohort”). From the IEO cohort, we randomly selected a subcohort of 672 patients corresponding with ∼27% of the entire cohort (the “subcohort” of Table S3). The subcohort contained 585 patients free of distant metastasis and 87 patients with distant metastasis. (B) We verified that the subcohort was representative of the IEO cohort. Indeed, there were no significant differences in the characteristics of patients between the patients in the subcohort and the excluded ones (“not in subcohort” as in Table S3). (C) To yield the final case cohort, we added to the subcohort all the remaining patients from the IEO cohort who experienced distant metastasis within 10 yr (an additional 218 patients for whom RNA was available). Therefore, the final case cohort included 305 patients with distant metastasis and 585 patients that were free of distant metastasis at 10 yr of followup. The primary endpoint of the analyses was distant metastasis, defined as the time from surgery to the appearance of distant metastases or death from BC as first event.

The association between Numb mRNA levels (quintile groups; Fig. S4 D) and the risk of distant metastasis at 10 yr after surgery was evaluated with the univariate and multivariable pseudolikelihood modification of Cox proportional hazards regression discussed by Self and Prentice (1988); Fig. 10, F and G). The robust variance estimates (Lin and Wei, 1989; Barlow, 1994) was used to construct Wald-type confidence intervals and significance tests for these parameter estimates. These analyses can be performed using widely available software (e.g., the cch function in the R survival package). All p-values are two-sided with a significance level of 0.05.

### P53 IHC on clinical samples

p53 IHC was performed in a Bond Max Automated Immunohistochemistry Vision Biosystem (Leica Microsystems) using the Bond Polymer Refine Detection kit (DS9800; Leica Biosystems). 3-µm-thick sections were prepared from formalin-fixed paraffin-embedded TMA tissue blocks, deparaffinized, pretreated with epitope retrieval solution 2 (pH9; Leica Biosystems) at 100°C for 20 min, and then incubated for 30 min with primary antibody (p53 DO-1; Santa Cruz Biotechnology, Inc.) diluted in Bond Primary Antibody Diluent (AR9352; Leica Biosystems) at final concentration of 40 ng/ml. All TMA slides were acquired with an Aperio ScanScope system (Leica Microsystems) and reviewed by a pathologist. For each core, 500 cells were counted, and the percentage of nuclear-stained positive cells was scored.

### Protein expression and purification

As a general procedure, GST-fusion proteins were expressed in BL21 Rosetta *Escherichia coli* cells by 6–8 h induction at 20°C with 0.3 mM IPTG. Cells were lysed in 100 mM Tris-HCl, pH 7.6, 0.3 M NaCl, 10% glycerol, 0.5 mM EDTA, 1 mM DTT, 0.2 mg/ml lysozyme, and protease inhibitors, and then were sonicated and cleared for 30 min at 20,000 *g*. The cleared lysates were incubated with Glutathione Sepharose 4 Fast-Flow beads (GE Healthcare), and the retained proteins were cleaved with PreScission protease overnight at 4°C to remove the GST tag (when required). The cleaved material was eluted from the beads in a desalting buffer consisting of 20 mM Tris-HCl, pH 7.6, 40 mM NaCl, 5% glycerol, 1 mM DTT, and 0.5 mM EDTA and then was loaded on a Resource-Q column (GE Healthcare).

His-tagged proteins were expressed in BL21 Rosetta pLysS *E. coli* cells by 3 h induction at 30°C with 1 mM IPTG. Cells were lysed in 100 mM Tris-HCl, pH 7.6, 0.3 M NaCl, 10% glycerol, 0.2 mg/ml lysozyme, 2 mM 2-mercaptoethanol, 20 mM imidazole, and protease inhibitors and then were sonicated and cleared for 30 min at 20,000 *g*. The cleared lysates were incubated with NTA beads (QIAGEN), and retained proteins were eluted in 50 mM Tris-HCl, pH 7.6, 0.15 M NaCl, and 200 mM imidazole and then dialyzed overnight at 4°C in 50 mM Tris-HCl, pH 7.6, 0.15 M NaCl, 0.5 mM EDTA, and 1 mM DTT with the addition of PreScission protease to remove the His tag when required. The cleaved material was loaded on a Superdex-200 column (GE Healthcare) equilibrated with the same buffer. The peak fractions were pooled and concentrated to ∼10 mg/ml using Vivaspin concentrators (Sartorius), flash frozen in liquid nitrogen, and stored at −80°C.

To purify Numb-PTBi for crystallization experiments, a Superdex-200 column was equilibrated in 20 mM sodium citrate, pH 5.5, 0.15 M NaCl, 0.5 mM EDTA, and 1 mM DTT. The peak fractions were pooled, supplemented with a 1.3 molar excess of synthetic GPpY peptide (AYIGPpYL; United Biosystems), and concentrated to ∼25 mg/ml.

For NMR experiments, proteins were prepared after the purification steps in a 20-mM sodium phosphate buffer, pH 6.8, 0.1 M NaCl, 0.02% sodium azide, and 1 mM DTT. To produce the proteins labeled with [^15^N] and [^13^C], the bacteria were grown in M9 medium with [^15^N]H_4_Cl and [^13^C]glucose as the sole nitrogen and carbon sources, respectively.

### Biochemical studies

Procedures for immunofluorescence (IF), immunoblotting (IB), and immunoprecipitation (IP) were as described previously (Pece et al., 2004) according to standard procedures.

For pulldown assays, GST-Mdm2 fragments (1 µM) were immobilized onto GSH beads and incubated for 2 h at 4 **°**C with Numb fragments at the indicated concentrations in 10 mM Hepes, pH 7.5, 0.15 M NaCl, 5% glycerol, and 0.1% Tween-20. After washing, proteins retained on beads were separated by SDS-PAGE and detected by Coomassie staining.

For the in vivo p53 ubiquitination assay (Fig. 8, A and B), 10-cm plates of H1299 cells were transfected with 1 µg p53-coding vector, 1.3 µg Mdm2-coding vector, 5 µg His6-Ub–coding vector, 0.1 µg GFP-coding vector (to monitor transfection efficiency), and 4 µg Numb-coding vector (2, 4, and 6 µg in Fig. 8 A). After 48 h, ubiquitin conjugates were purified as described previously by Gabellini et al. (2003). For in vitro p53 ubiquitination assay, the reaction mixture contained the following purified enzymes: 60 nM E1, 1 µM UbcH5B, 0.15 µM GST-Mdm2, 70 nM p53, and 50 µM ubiquitin (p53 was generated in-house; all other enzymes were purchased from Boston Biochem). The reaction was assembled in ubiquitination buffer (B-71; Boston Biochem) supplemented with Mg-ATP solution (B-20; Boston Biochem). When appropriate, 5 µM of the proteins indicated in Fig. 8 C were added to the reaction mixture. Reactions were incubated at 37°C for 10 min followed by addition of Laemmli buffer and 50 µM DTT. The Mdm2 inhibitor RG7112 was used at a 10-µM final concentration.

### Structure determination

Crystallization experiments of Numb-PTBi in complex with the GPpY peptide were performed at the IEO Crystallography Unit. Diffraction-quality crystals were obtained by manual optimization of the initial conditions in hanging drops at 20°C, mixing 1 µl of protein with 0.5 µl of reservoir containing 25% PEG 3350, 0.2 M lithium sulfate, and 0.1 M Bis-Tris, pH 6. Before equilibration, drops were further supplemented with 0.5 µl of silver bullets (0.33% [wt/vol] anthrone, 0.33% [wt/vol] Congo red, 0.33% [wt/vol] *N*-(2-acetamido)-2-aminoethanesulfonic acid, and 20 mM Hepes, pH 6.8; n. 13) to obtain F222 crystals or with 0.5 µl of silver bullets (4 mM calcium chloride dihydrate, 4 mM magnesium chloride hexahydrate, 4 mM manganese(II) chloride tetrahydrate, 4 mM zinc chloride, and 20 mM Hepes, pH 6.8; n. 41) and 0.3 µl of additive screen (100 mM l-proline; n. 26; Hampton Research) to obtain P2_1_2_1_2_1_ crystals. Crystals were cryoprotected by soaking in reservoir buffer supplemented with 20% glycerol and flash-frozen in liquid nitrogen. X-ray diffraction data were collected at beamline X06DA (PXIII) of the Swiss Lightsource and beamline ID23-2 at the European Synchroton Radiation Facility. The crystals belonged to space group F222 or P2_1_2_1_2_1_. Initial phases were obtained by molecular replacement using the PTB domain of mouse Numb as a search model (PDB ID 3FOW). The model was then completed using iterative cycles of manual building and restrained refinement with PHENIX (Adams et al., 2010). The four copies of the Numb-PTBi–GPpY complex present in the asymmetric unit (a.s.u.) of the F222 space group and the six copies of a.s.u of the P2_1_2_1_2_1_ space group were identical except for the insert region, which was not visible in five of the copies. Data processing and refinement statistics are shown in Table S1.

For the superposition, we used the PTBi copies present in the two crystal forms in which we crystallized Numb-PTBi (P2_1_2_1_2_1_ and F222) and the structure of mouse PTBo (PDB ID 3F0W). The superposition was performed with the online server Rapido.

### Accession numbers

The coordinates and the structure factors have been deposited to the Protein Data Bank under accession numbers 5NJJ and 5NJK.

### Fluorescence polarization (FP)

FP measurements were performed on an Infinite F200 plate reader (Tecan). In vitro rhodaminated-Mdm2^216–302^ (50 nM) was incubated with increasing concentrations of the indicated ligand in 10 mM Tris-HCl, pH 7.5, 0.15 M NaCl, 0.5 mM EDTA, and 1 mM DTT. The corresponding K_D_ were calculated by fitting the FP curves in Prism (GraphPad Software).

### NMR spectroscopy

Spectra were recorded on Bruker AV500, AV600, or AV 800 spectrometers with cryogenic [^1^H], [^15^N], and [^13^C] triple cryogenic inverse triple resonance probeheads at 298 K. Data were processed using NMRPIPE (Delaglio et al., 1995) and analyzed with Collaborative Computational Project NMR Analysis (Vranken et al., 2005). For NMR experiments, protein samples were in 90%/10% H_2_O/D_2_O, 20 mM sodium phosphate, 200 mM NaCl, 1 mM DDT, and 0.02% NaN_3_, pH 6.8. Backbone assignments were determined through the standard backbone resonance experiments HNCA, HNCACB, CBCA(Co)NH, HNCO, and HN(CA)CO (Sattler et al., 1999). Aromatic spectra were obtained using an HSQC-TROSY base experiment (Pervushin et al., 1998) centered at 122.5 ppm with a 16.6-ms constant time delay to eliminate splitting from aromatic C–C couplings. Aromatic assignments were obtained from [^15^N]-edited and [^13^C]-edited NOESY centered on aromatics (122.5 ppm), (HB)CB (CGCD)HD, and (HB)CB(CGCDCE)HE experiments (Yamazaki et al., 1993). For Mdm2 experiments, to avoid precipitations, both proteins were diluted to ∼50 µM combined and then concentrated to 200 µM. CSP data were obtained from 2D [^15^N-^1^H] or [^13^C-^1^H]-CT correlation experiments. The CSP was calculated as

ΔδN−H=(ΔδH1*10)2+(Δδ|15N)2.

Numb^PTBi^ selectively labeled with SAIL-Phe (Kainosho et al., 2006; Takeda et al., 2010) was prepared using the *E. coli* strain AB2826 (DE3), which is auxotrophic for aromatic amino acids according to the recently described protocol (Yang et al., 2015). SAIL-Phe–labeled Numb^PTBi^ was isolated by binding to NTA beads (QIAGEN) and then purified on a Superdex-200 column.

### Imaging studies

The images in Fig. 8 A were acquired by confocal analyses performed with a TCS SP5 acousto optical bream splitter microscope system equipped with HyD (high-quantum-efficiency hybrid detector) and photomultiplier tube detectors (Leica Microsystems). Images of the fixed samples were acquired at RT using a 40× 1.3 NA oil immersion objective under the control of LAS AF software (Leica Microsystems).

The images of Fig. 9 C were acquired by widefield microscopy with a BX63 microscope equipped with an XM10 detector (Olympus). Images of the fixed samples were acquired at RT using a 20× 0.75 NA objective under the control of MetaMorph software (Molecular Devices).

Images were analyzed with tools available via ImageJ/Fiji software (National Institutes of Health) and Photoshop (Adobe).

### Online supplemental material

Supplemental data for this article include four additional figures and two tables showing an electron density map of the PTBi insert (Fig. S1); additional experiments showing how the PTBi binds Mdm2 in a noncanonical manner (Fig. S2); additional characterizations of the PTBi–Mmd2 interface (Fig. S3); specificity of the RT-qPCR Numb assays (Fig. S4 A); Mdm2 titration for the p53 in vivo ubiquitination assay (Fig. S4 B); RT-qPCR of the MCF-10A cells used in the experiment of Fig. 9 A (Fig. S4 C); range of mRNA levels into the quintile groups for the NUMB isoforms analyzed in Fig. 10 (F and G; Fig. S4 D); p53 immunohistochemistry (Fig. S4, E and F); data collection of the Numb-PTBi–GPpY crystal structure (Table S1); distribution of adjuvant treatment received by the patients of the cohort stratified according the subtype, p53 status, and Numb-1/2 or -3/4 status (Table S2); and distribution of patient characteristics in the random subcohort compared with the entire IEO cohort and the not-in-subcohort (Table S3).

## Supplementary Material

Supplemental Materials
