# Supplementary material for: A Numb–Mdm2 fuzzy complex reveals an isoform-specific involvement of Numb in breast cancer
Source: J Cell Biol. 2018 Feb 5;217(2):745–62. doi: 10.1083/jcb.201709092 (PMC5800818; doi:10.1083/jcb.201709092)
Supplement: Supplemental Materials [file JCB_201709092_sm.pdf]

Colaluca et al., https://doi.org/10.1083/jcb.201709092

JCB

JOURNAL OF CELL BIOLOGY

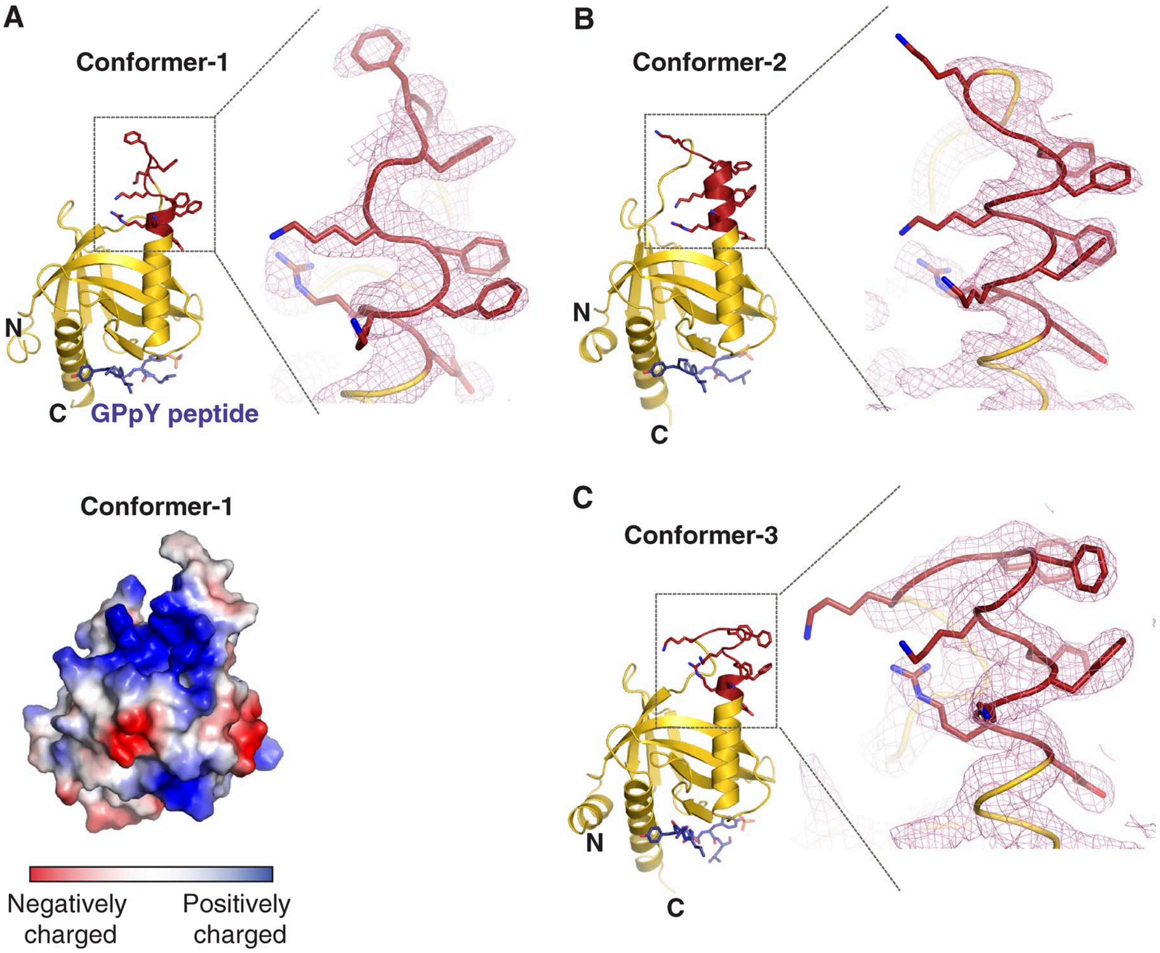

Figure S1. **Electron density maps of the PTBi inserts encoded by Ex3. (A–C)** Left: Ribbon models of the three independent Numb-PTBi conformers present in the a.s.u. depicted with the same color code and orientation as in Fig. 3 A. Right: Close-up views with $3F_{obs}$-$2F_{calc}$ electron density maps contoured at 1.0 σ level around the Numb-PTBi inserts. The bottom part of A shows a surface representation of conformer-1 of Numb-PTBi colored by electrostatic potential.

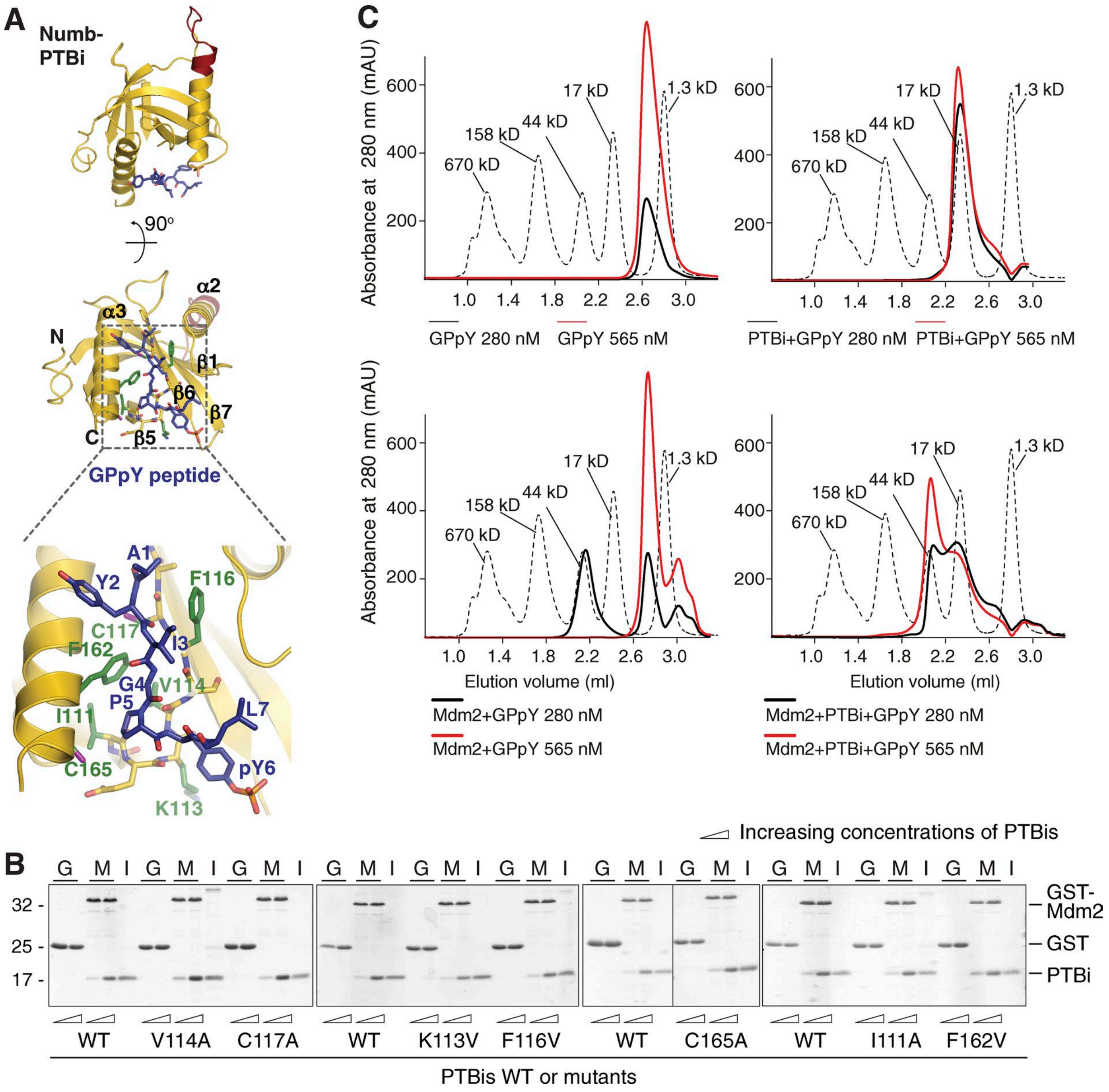

Figure S2. **The PTBi binds to the acidic domain of Mdm2 in a noncanonical manner. (A–C)** The overall similarity of PTBo and PTBi and the fact that the Ex3-coded region is completely distinct from the peptide-binding region strongly suggest that the Mdm2–PTBi interaction must reside in a novel surface, possibly coinciding with the Ex3-encoded sequence. This contention was initially supported by mutagenesis research in which we engineered seven PTBi variants (A) harboring mutations known to impair Numb peptide binding (Zwahlen et al., 2000); none of these mutants displayed altered binding to Mdm2 (B). Consistently, SEC analysis (C) revealed that Mdm2 enters a trimeric complex with the Numb PTBi and GPpY with unaltered affinity. Thus, Mdm2 binds to a surface other than the peptide-binding cleft of the PTBi. **(A)** Cartoon representation of Numb-PTBi viewed at 90° rotation to evidence the peptide-binding groove. The GPpY peptide is displayed in blue. The strand β5 of Numb-PTBi and the GPpY peptide, which engage in β sheet interactions, are displayed in balls and sticks. Bottom: enlarged view of the Numb-PTBi peptide-binding groove with residues of Numb contacting the GPpY ligand displayed in green. **(B)** Pulldown experiments with the GST proteins indicated on top (G, GST; M, GST-Mdm2$^{216–302}$; 1 µM) incubated with increasing concentrations (1 µM and 5 µM) of WT or mutant PTBis (mutated amino acids are indicated, and proteins were bacterially expressed and eluted from NTA beads). The mutants were engineered in residues important for the recognition of the GPpY phosphopeptide. Species retained on GSH beads were separated by SDS-PAGE and Coomassie stained. I, input PTBs. All lanes shown in the third panel were run on a single gel; black lines indicate excision of intervening lanes. Molecular masses are given in kilodaltons. **(C)** SEC elution profiles (Superdex-200 column) of 300 µM fluorescein-labeled GPpY peptide in isolation or incubated with 300 µM Numb-PTBi, Mdm2$^{216–302}$, or both. Traces were recorded at 280 nm (black) and 565 nm (red) to distinguish the fluorescein-labeled GPpY peptide. Incubation of the three components results in the formation of a trimeric Numb-PTBi–GPpY–Mdm2$^{216–302}$ complex eluting in proximity to the 44-kD molecular weight marker.

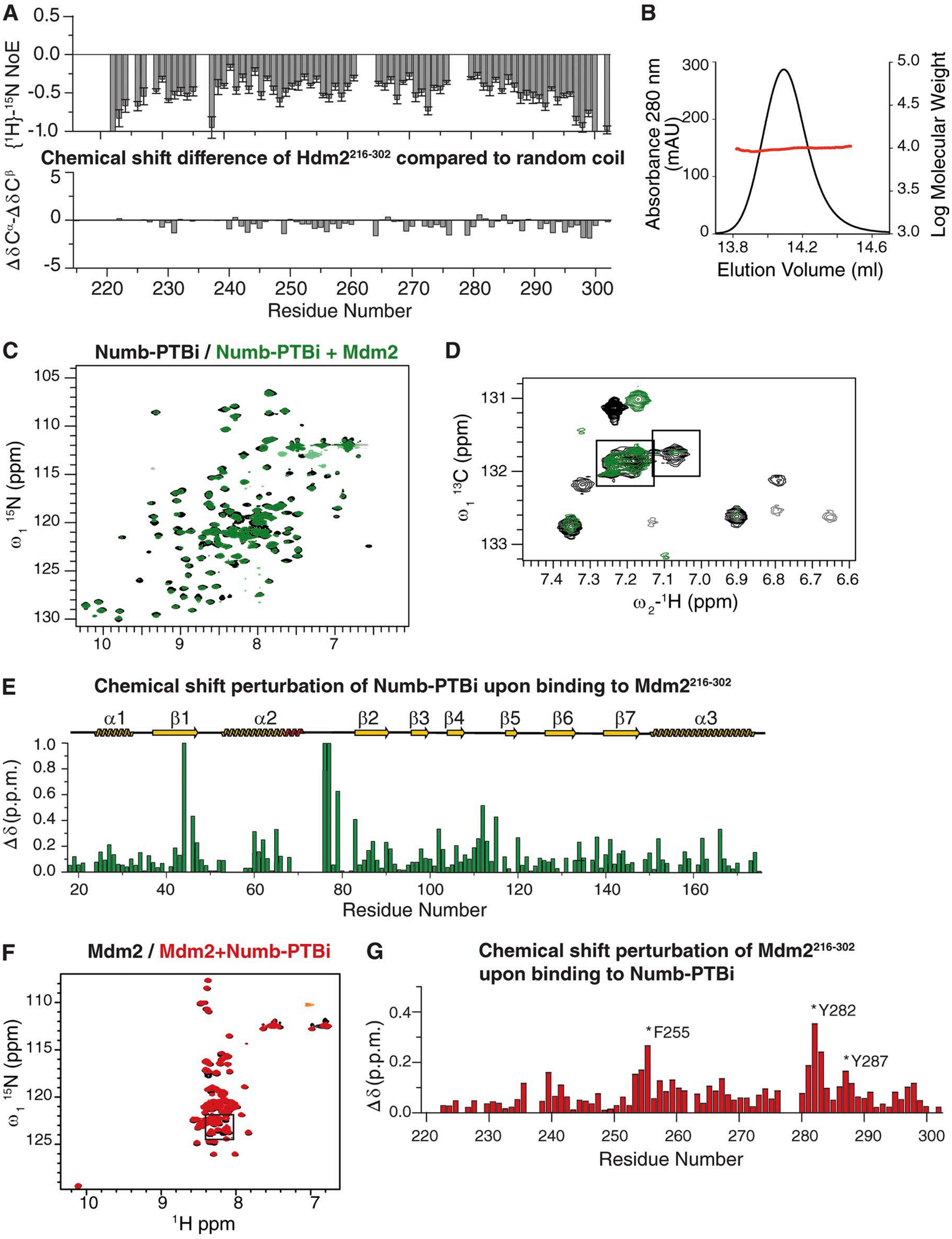

Figure S3. **Additional characterizations of the PTBi–Mdm2 interface. (A)** Top: [1H][15N] heteronuclear nuclear Overhauser effect (NOE) experiments (Farrow et al., 1994) recorded on [15N]-labeled Mdm2$^{216–302}$. A positive NOE of 0.8 is expected for structured regions, whereas values <0 indicate regions with high internal flexibility at subnanosecond timescales. Error bars indicate uncertainties based on the noise level in the NMR spectra for the NOEs derived using error propagation. Bottom: secondary chemical shifts derived from the difference of [13Cα] and [13Cβ] chemical shifts recorded for Mdm2$^{216–302}$ indicate a mainly random coil conformation. **(B)** Static light-scattering profile of Mdm2$^{216–302}$ showing a molecular mass of ~9 kD along the peak as expected for a monomeric species. **(C)** Superposition of [1H][15N] HSQC spectra of [15N]-labeled Numb-PTBi free (black) or in the presence of 1.1 equivalents of Mdm2$^{216–302}$ (green). **(D)** Superposition of [1H][13C] correlation spectra of Phe-SAIL–labeled Numb-PTBi free (black) or in the presence of 1.2 equivalents of Mdm2$^{216–302}$ (green) showing the signals of aromatic residues. Boxes correspond with the insets presented in Fig. 4 C. **(E)** Overall CSP changes of Numb-PTBi [1H][15N] HSQC spectra for binding to Mdm2$^{216–302}$. **(F)** Superposition of [1H][15N] HSQC spectra of Mdm2$^{216–302}$ free (black) or in the presence of 1.1 equivalents of Numb-PTBi (red). The box corresponds with the inset in Fig. 5 A. **(G)** Summary of the CSPs observed in [1H][15N] HSQC spectra of Mdm2$^{216–302}$ binding Numb-PTBi versus Mdm2 residues. Asterisks indicate major contact residues with PTBi as validated in Fig. 6 (B and F).

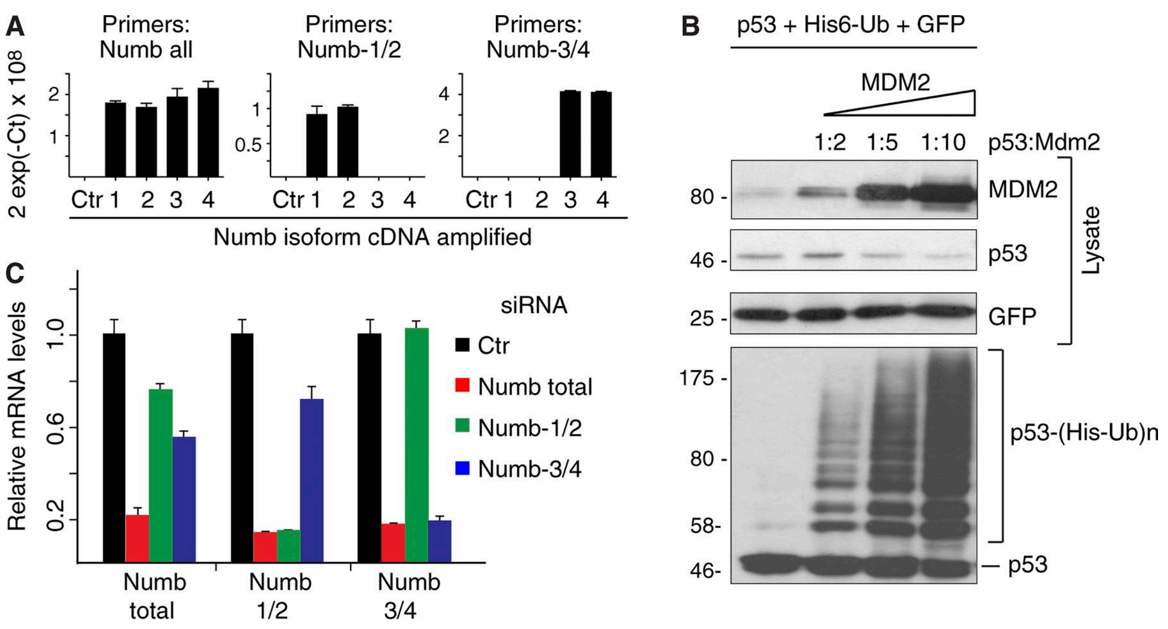

D

Quintile	N	Numb 1/2 (min; max)	N	Numb 3/4 (min; max)
1	178	29.2; 32.8	178	28.5; 34.6
2	178	28.7; 29.2	178	28.1; 28.5
3	178	28.3; 28.7	178	27.8; 28.1
4	178	27.9; 28.3	178	27.5; 27.8
5	178	23.0; 27.9	178	25.7; 27.5

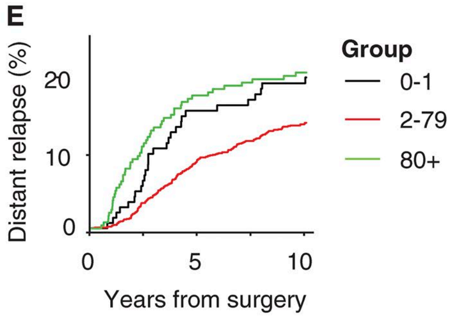

HR (95% CI)

0-1 *vs*. 2-79 : 1.54 (1.04; 2.29), p=**0.032**

0-1 *vs*. 80+ : 0.92 (0.58; 1.46), p=0.72

80+ *vs*. 2-79 : 1.68 (1.23; 2.29), p=**0.001**

F

p53 group	N	N. events
0-1	143	28
2-79	1463	204
80+	239	49
NA	608	38

Figure S4. **Additional data relative to experiments described in Figs. 7, 8, 9, and 10. (A)** Specificity of the quantitative RT-PCR Numb assays. pcDNA vectors containing the coding sequence of the four Numb isoforms (as shown at the bottom) were amplified with the primers indicated at the top. Empty pcDNA (Ctr) was used as a negative control. **(B)** Mdm2 titration for the p53 in vivo ubiquitination assay. Ubiquitination assays in H1299 cells transfected with p53, His-Ub, GFP (transfected as an internal normalizer for the efficiency of transfection), and increasing concentrations of Mdm2 (at the indicated ratio with respect to p53). Lysates were subjected to IB as indicated. p53 level reduction by Mdm2 is appreciable only using a large excess of Mdm2, explaining why in Fig. 8 (A and B), only the p53 ubiquitination is appreciable (a ratio Mdm2/p53 1.3:1 was used in these competition ubiquitination assays). Molecular masses are given in kilodaltons. **(C)** RT-qPCR of the MCF-10A cells (silenced as shown) used in the experiments of Fig. 9 A with primers specifically amplifying the indicated Numb isoforms. Each target was analyzed in triplicate, and the means ± SD are shown. **(D)** Range of mRNA levels into the quintile groups for the indicated Numb isoforms expressed as $Cq_{normalized}$ values. Max, maximum $Cq_{normalized}$; Min, minimum $Cq_{normalized}$.; N, number of patients. The group indicated as "Low" in Fig. 10 F corresponds with quintile 1, and the group "High" corresponds with the quintiles 2–5. **(E and F)** p53 expression by IHC analysis has been largely used as a surrogate for mutational analysis (Alsner et al., 2008). Generally, a strong staining is regarded as indicative of a missense mutation of the *p53* gene; it has been also proposed that absence of expression by IHC may indicate a nonsense mutation, leading to formation of a truncated protein (Alsner et al., 2008; Yemelyanova et al., 2011). We screened our entire cohort of 2,453 BC cases (the IEO cohort) for p53 by IHC. Patients were classified according to the p53 nuclear staining (see also the p53 IHC on clinical samples section of Materials and methods) into three groups: 0–1, 0–1% positive nuclei (indicative of complete loss of p53 protein/nonsense mutations); 2–79, 2–79% positive nuclei (indicative of WT levels of p53); 80+, positive p53 nuclei ≥80% (indicative of missense mutations of p53). **(E)** Cumulative incidence of distant metastasis in the consecutive cohort of 2,453 BC patients clustered in three groups according to the p53 level in IHC. The hazard ratio (HR) comparison among the p53 groups (with the p-values) is shown and was estimated with a univariate Cox proportional hazards model. High-p53 IHC staining (80+ group) or loss of p53 IHC signal (0–1 group) are indicative of a poor prognosis, suggesting that p53 IHC can be used as a reliable indicator of the presence of mutated p53 protein. CI, confidence interval. **(F)** Distribution of the 2,453 BC patients into the p53 IHC groups. N, number of patients; N. events, number of patients with distant metastasis; NA, p53 IHC not available. The percentage of p53-mutated samples (groups 0–1 and 80+ combined) in the cohort (considering the samples with an available p53 IHC classification) is ∼20%, in accordance with the percentage of p53 mutations described in BC (Gasco et al., 2002).

Table S1. **Crystal structure of Numb-PTBi in complex with GPpY: Data collection and refinement statistics**

**Crystal forms**	**F222**	**$P2_12_12_1$**
**Data collection**		
Space group	F222	$P2_12_12_1$
Cell dimensions		
a, *b*, *c* (Å)	113.8, 120.4, 237.4	75.3, 81.9, 235.7
α, β, γ (°)	90.0, 90.0, 90.0	90.0, 90.0, 90.0
Resolution (Å)	40.0 - 2.7 (2.8 - 2.7)[a]	40.0 - 3.13 (3.24 - 3.13)
R_{sym} or R_{merge} (%)	9.66 (134.3)	15.6 (99.3)
$I / \sigma I$	18.4 (2.2)	9.5 (1.6)
Completeness (%)	99.6 (98.6)	99.4 (98.1)
Redundancy	13.2 (12.9)	4.9 (4.8)
**Refinement**		
Resolution (Å)	40.0 - 2.7	40.0 - 3.13
No. reflections	22,466	26,321
R_{work} / R_{free}	26.8 / 30.3	22.9 / 26.0
No. atoms		
Protein	4725	6972
Ligand/ion	5	10
Water	1	2
B-factors		
Protein	81.4	66.6
Ligand/ion	81.9	68.2
Water	69	57.2
R.m.s.[b] deviations		
Bond lengths (Å)	0.012	0.015
Bond angles (°)	1.87	1.85

[a]Values in parentheses are for the highest-resolution shell.
[b]root mean square.

Table S2. **Distribution of adjuvant treatment received by the patients of the cohort stratified according the subtype, p53 status, and Numb-1/2 or -3/4 status**

Group	Adjuvant treatment	N (%Col)			P[a]	N (%Col)		P
		All	Numb-1/2 low	Numb-1/2 high		Numb-3/4 low	Numb-3/4 high	
**Entire Cohort**								
All	All	890 (100)	178 (20)	712 (80)		178 (20)	712 (80)	
	Nil[b]	24 (2.7)	5 (20.8)	19 (79.2)	0.422	2 (8.3)	22 (91.7)	0.216
	HT[c]	262 (29.4)	56 (21.4)	206 (78.6)		46 (17.6)	216 (82.4)	
	CT[d]	130 (14.6)	19 (14.6)	111 (85.4)		31 (23.8)	99 (76.2)	
	HT+CT	474 (53.3)	98 (20.7)	376 (79.3)		99 (20.9)	375 (79.1)	
p53 Mut	All	184 (100)	32 (17.4)	152 (82.6)		51 (27.7)	133 (72.3)	
	Nil	6 (3.3)	0 (0)	6 (100)	0.578	0 (0)	6 (100)	0.174
	HT	16 (8.7)	4 (25)	12 (75)		7 (43.7)	9 (56.2)	
	CT	73 (39.7)	12 (16.4)	61 (83.6)		22 (30.1)	51 (69.9)	
	HT+CT	89 (48.4)	16 (18)	73 (82)		22 (24.7)	67 (75.3)	
p53 WT	All	611 (100)	128 (20.9)	483 (79.1)		108 (17.7)	503 (82.3)	
	Nil	16 (2.6)	5 (31.2)	11 (68.7)	0.506	2 (12.5)	14 (87.5)	0.594
	HT	214 (35)	46 (21.5)	168 (78.5)		33 (15.4)	181 (84.6)	
	CT	43 (7)	6 (14)	37 (86)		7 (16.3)	36 (83.7)	
	HT+CT	338 (55.3)	71 (21)	267 (79)		66 (19.5)	272 (80.5)	
**Luminal cases**								
All	All	666 (100)	149 (22.4)	517 (77.6)		120 (18)	546 (82)	
	Nil	16 (2.4)	5 (31.2)	11 (68.7)	0.836	0 (0)	16 (100)	0.199
	HT	247 (37.1)	53 (21.5)	194 (78.5)		41 (16.6)	206 (83.4)	
	CT	18 (2.7)	4 (22.2)	14 (77.8)		3 (16.7)	15 (83.3)	
	HT+CT	385 (57.8)	87 (22.6)	298 (77.4)		76 (19.7)	309 (80.3)	
p53 Mut	All	84 (100)	17 (20.2)	67 (79.8)		21 (25)	63 (75)	
	Nil	2 (2.4)	0 (0)	2 (100)	0.547	0 (0)	2 (100)	0.215
	HT	14 (16.7)	4 (28.6)	10 (71.4)		6 (42.9)	8 (57.1)	
	CT	4 (4.8)	0 (0)	4 (100)		0 (0)	4 (100)	
	HT+CT	64 (76.2)	13 (20.3)	51 (79.7)		15 (23.4)	49 (76.6)	
p53 WT	All	519 (100)	118 (22.7)	401 (77.3)		88 (17)	431 (83)	
	Nil	13 (2.5)	5 (38.5)	8 (61.5)	0.424	0 (0)	13 (100)	0.23
	HT	205 (39.5)	44 (21.5)	161 (78.5)		31 (15.1)	174 (84.9)	
	CT	12 (2.3)	4 (33.3)	8 (66.7)		3 (25)	9 (75)	
	HT+CT	289 (55.7)	65 (22.5)	224 (77.5)		54 (18.7)	235 (81.3)	

p53 status (when available) has been determined by IHC, as discussed also in Fig. S4, (E and F). Numb-1/2 and -3/4 low and high groups have been defined in Fig. 10 F.
[a]P value from χ^2 test.
[b]No adjuvant therapy.
[c]Adjuvant hormonal therapy.
[d]Adjuvant chemotherapy.

Table S3. **Distribution of patient characteristics in the random subcohort (*n* = 672) compared with the entire IEO cohort (All; *n* = 2,453) and the not-in-subcohort (*n* = 1,781)**

Variable	All	Not in subcohort	Subcohort	P[a]
**All**	2,453	1,781	672	
**Age at surgery**				0.06
<50	969 (39.5)	683 (38.3)	286 (42.6)	
≥50	1,484 (60.5)	1,098 (61.7)	386 (57.4)	
**Histology**				0.43
Ductal	1,960 (79.9)	1,430 (80.3)	530 (78.9)	
No Ductal	493 (20.1)	351 (19.7)	142 (21.1)	
**pT**				0.06
pT1	1,616 (65.9)	1,199 (67.3)	417 (62.1)	
pT2	756 (30.8)	521 (29.3)	235 (35.0)	
pT3	63 (2.6)	47 (2.6)	16 (2.4)	
pT4	18 (0.7)	14 (0.8)	4 (0.6)	
**Positive lymph nodes**				0.16
None	1,207 (49.2)	893 (50.1)	314 (46.7)	
1+	1,187 (48.4)	842 (47.3)	345 (51.3)	
NA[b]	59 (2.4)	46 (2.6)	13 (1.9)	
**Grade**				0.53
1-2	1,537 (62.7)	1,126 (63.2)	411 (61.2)	
3	859 (35.0)	619 (34.8)	240 (35.7)	
NA	57 (2.3)	36 (2.0)	21 (3.1)	
**PVI**[c]				0.16
Absent	1,681 (68.5)	1,235 (69.3)	446 (66.4)	
Present	772 (31.5)	546 (30.7)	226 (33.6)	
**ER**[d]				0.47
ER = 0	375 (15.3)	278 (15.6)	97 (14.4)	
ER > 0	2,078 (84.7)	1,503 (84.4)	575 (85.6)	
**ER/PgR**[e]				0.56
Nonexpressed (both 0)	341 (13.9)	252 (14.1)	89 (13.2)	
Expressed (ER > 0 or PgR > 0)	2,112 (86.1)	1,529 (85.9)	583 (86.8)	
**HER2 status**				0.45
Negative	1,935 (78.9)	1,356 (76.1)	579 (86.2)	
Positive	264 (10.8)	191 (10.7)	73 (10.9)	
NA	254 (10.4)	234 (13.1)	20 (3.0)	
	**Mean (SD)**			**P**[f]
**Ki-67**	24.0 (16.7)	23.8 (16.7)	24.5 (16.5)	0.34

Differences in the distribution of clinical–pathological features between subcohort and not-in-subcohort group were evaluated by the χ^2 test or *t* test. The middle three columns show the number of patients along with percentages of their respective columns in parentheses except for the last row, which shows means along with SD in parentheses.
[a]P-value derived from χ^2 test.
[b]Not available.
[c]Perivascular invasion.
[d]Estrogen receptor.
[e]Progesterone receptor.
[f]P-value derived from *t* test.

References

Alsner, J., V. Jensen, M. Kyndi, B.V. Offersen, P. Vu, A.L. Børresen-Dale, and J. Overgaard. 2008. A comparison between p53 accumulation determined by immunohistochemistry and TP53 mutations as prognostic variables in tumours from breast cancer patients. *Acta Oncol.* 47:600–607. https://doi.org/10.1080/02841860802047411

Farrow, N.A., R. Muhandiram, A.U. Singer, S.M. Pascal, C.M. Kay, G. Gish, S.E. Shoelson, T. Pawson, J.D. Forman-Kay, and L.E. Kay. 1994. Backbone dynamics of a free and phosphopeptide-complexed Src homology 2 domain studied by 15N NMR relaxation. *Biochemistry.* 33:5984–6003. https://doi.org/10.1021/bi00185a040

Gasco, M., S. Shami, and T. Crook. 2002. The p53 pathway in breast cancer. *Breast Cancer Res.* 4:70–76. https://doi.org/10.1186/bcr426

Yemelyanova, A., R. Vang, M. Kshirsagar, D. Lu, M.A. Marks, IeM. Shih, and R.J. Kurman. 2011. Immunohistochemical staining patterns of p53 can serve as a surrogate marker for TP53 mutations in ovarian carcinoma: an immunohistochemical and nucleotide sequencing analysis. *Mod. Pathol.* 24:1248–1253. https://doi.org/10.1038/modpathol.2011.85

Zwahlen, C., S.C. Li, L.E. Kay, T. Pawson, and J.D. Forman-Kay. 2000. Multiple modes of peptide recognition by the PTB domain of the cell fate determinant Numb. *EMBO J.* 19:1505–1515. https://doi.org/10.1093/emboj/19.7.1505
